# A Treatise on the Venereal Disease and Its Varieties

**Published:** 1833-10-01

**Authors:** 


### VIII.

A TREATISE ON THE VENEREAL DISEASE AND ITS VARIETIES. By *William Wallace*, M.R.I.A. &c. Surgeon to the Jervis Street Infirmary, Dublin, and to the Infirmary established in that City for the Treatment of Cutaneous Diseases, including Venereal Diseases. Octavo, pp. 382. London, 1833.

[Second Article.]

IN our last Number, we commenced an analytical review of a work on the Venereal Disease, by Mr. Wallace, of Dublin. That notice was limited to the introductory portion of the volume, and we found occasion to join issue with Mr. Wallace on many theoretical and doctrinal points. We remarked, however, that when we arrived at the descriptive portion, we should probably find occasion to express our satisfaction at the closeness of observation and fidelity of description.

Mr. Wallace arranges the various primary symptoms, produced by the application of the venereal poison, under the heads of—1. Primary syphilis. 2. Degenerations of primary syphilis.

"Primary syphilis is characterized by uniformly exhibiting during its progress a certain series of destructive actions, accompanied, or else sooner or later followed by a corresponding series of reparative actions:—the latter series always bearing a fixed relation to the former. It will also be found, that each of these series of actions is composed of a minor series; which, in like manner, bear a regular and fixed proportion to each other.

But, on the other hand, the degenerations of primary syphilis do not exhibit any fixed relations or proportions between their destructive actions, as compared with their actions of protection and reparation, nor between the minor actions, which respectively compose each of these series ; and it will be found, that in each of these degenerate forms of primary syphilis, one or more of the essential or characteristic features of the regular disease exist, in a state of monstrosity. Thus,—phlogosis, inflammation, ulceration, induration or interstitial deposition, granulation, cicatrization, &c. &c., are always exhibited in certain proportions by the regular form ; but, in the degenerate forms, one or more of these actions are sometimes much increased, and at other times they are equally deficient. In short, all natural proportion is wanting. Thus the irregular forms of primary syphilis are sometimes characterized by great induration, at other times by great ulceration, and at others by the very opposite state, as exuberant granulation." 61.

This seems too mathematically precise to lead us to augur much in its favour. On this Mr. Wallace founds his classification—an ascending and a descending scale, his genuine syphilis constituting the centre of his system. All the degenerate forms of primary syphilis he refers to two divisions—those of the first being characterized principally by irregularity in the processes of ulceration or destruction, and those of the second, by irregularity in the processes of reparation.

The former division is composed of three varieties : in the first the destructive process is in excess,—phagedænic primary syphilis ; in the second the same process is deficient,—superficial primary syphilis ; and in the third it is wanting,—catarrhal primary syphilis.

The second division consists also of three varieties : the first is characterized by excess of interstitial deposition at the base of the ulcer,—indurated primary syphilis ; the second by excess of interstitial deposition at the circumference of the ulcer,—annular primary syphilis ; and third by an excess of granulation or deposition on the surface of the diseased part,—fungous primary syphilis :—

Hence there are the six following varieties of irregular primary syphilis :

1. Phagedænic primary syphilis.
2. Superficial primary syphilis.
3. Catarrhal primary syphilis.
4. Indurated primary syphilis.
5. Annular primary syphilis.
6. Fungous primary syphilis.

We cannot say much for the natural character of a classification, which makes phagedæna and gonorrhœa varieties of one division, and places in two opposite divisions the superficial and the fungous sores. The classification is, as a classification, a bad one, and no one, we are confident, can rise from the perusal of the introductory part of this volume with notions of the venereal disease in any respect clearer than, nay, with notions as clear as, when he sat down to its perusal. Nosological classifications in medicine are generally a failure. All that is wanted, is an arrangement which shall connect things in themselves similar, and enable us to study, with the least expense of time and trouble, the particular facts, and consolidated groups of facts that Nature furnishes. After all Mr. Wallace's laboured generalizations, we come at last to the investigation of the particular affections, gonorrhœa, indurated sore, phagedæna, &c. ; and his speculations have only succeeded in disturbing them from what appears their natural position, and placing them in forced and strained relations.

Before proceeding to the description of his specific form of syphilis, Mr. Wallace defines the terms which he proposes to employ. The *surface* of a sore is that which is undergoing the processes of ulceration and sloughing —the *areola* is so much of the skin surrounding an ulcer as may be diseased—the *base* is so much of the parts subjacent to or surrounding the surface of an ulcer and its areola, as may be in a diseased state—the *edge* is the line bounding the surface, or the line where the surface of the ulcer and the areola meet—the *margin of the ulcer* is that part immediately within the edge—the *margin of the areola,* or the *cutaneous margin of the ulcer,* is that part of the areola immediately surrounding the edge of the ulcer—the *rim, or border, of the ulcer* is formed by the union of the margins of the ulcer and of the areola. We cannot, for our parts, see the difference between the edge of the ulcer and the rim of the ulcer.

We have now arrived at the third chapter, in which is commenced the actual description of venereal diseases. We believe that, however we may have differed from Mr. Wallace on speculative points, we shall have the pleasure of bearing testimony to his accuracy of observation and practical acquaintance with his subject.

### I. PRIMARY SYPHILIS.

Mr. Wallace, as our readers are aware, assumes that this is the genuine form of syphilis. We shall look at it as a description of a sore, independently of any theoretical considerations. Mr. W. first delineates its characters, as it shews itself on the common skin of the penis.

### A. *Primary Syphilis on the Skin of the Penis.*

It most commonly occurs on the upper surface of the penis, and nearer its præputial than its pubic end, but it may be met in other situations. It is observed more frequently in the upper than in the lower ranks of society, probably from their want of cleanliness. We cannot condense the following description.

"Primary syphilis on the exterior of the penis commences by a red patch, about the size of a coriander seed, slightly deeper in colour at the centre than at the circumference. This red surface, which is of a dark hue, soon becomes a little elevated or tumid in the middle, and then exhibits somewhat the appearance of a papula on a diffused and inflamed base.

In the centre of this papula, a minute black point may be almost immediately observed, which in the course of another day is found to be a scab; and from the shining whitish-red appearance of the eminence, upon which this scab is placed, as well as from the presence of a whitish line which in general surrounds the scab, the tumidity seems to be produced by matter; yet the part does not in general exhibit a regular projecting pustular appearance, but rather an appearance as if the crust formed a covering for a cavity, which was seated much deeper in the skin than that of an ordinary pustule.

If on the following day the part be examined, the scab will probably appear depressed in its centre, and the surrounding cuticle slightly wrinkled. These changes result from the contents of the little abscess having been discharged, either by some accidental violence, which detached the edge of the scab and compressed the matter from under its surface, or in consequence of the patient having pressed the diseased part between his fingers and thumb; for at this period his attention is often attracted to the disease by a peculiar uneasiness, which excites a feeling that it would be relieved by compressing the part in which it is seated.

If no further mechanical violence be offered, and if the disease be allowed to remain unacted upon, the scab or crust, surrounded often by a whitish line inside a diffused and tumulated areola of a brownish-red colour, is gradually increased in thickness and extent, until the ulcerative process which is going on underneath this crust has ceased; and then the crust also ceasing to extend, the whitish line at its circumference disappears. The scab gradually becomes more dark-coloured, more contracted and hard, and now seems raised on an elevated basis. But after a time it again sinks, and then often appears depressed, particularly in the centre, almost below the level of the surrounding skin. When this last change has occurred, it soon falls off, and leaves the surface, upon which it had been placed, covered by a new skin of a livid colour and thin texture.

It seldom happens that the primary syphilitic ulcer remains encrusted, until perfect cicatrization has taken place; for, from various causes, its crust is most generally detached almost as soon as formed; and although it may fall or be removed in any stage of the ulcer, I shall suppose that it has been removed while the process of ulceration is still advancing, and before any action of reparation has commenced. Underneath it we then find a sore of a dusky yellowish white colour, often mingled with minute pinkish spots. In general, its edge appears nibbled as it were, or serrated, and of a white or whitish yellow colour, sometimes very lightly punctulated, or dotted with minute brown points; and often bounded on the inside by a line of white, soft, pulpy, or lardaceous looking matter." 68.

This ulcer appears to have penetrated through the whole thickness of the skin, and its surface, which often looks flat, seems to pass under the surrounding integument, as if overlapped by it. The areola surrounding the diseased surface often becomes more injected with blood than before the removal of the crust; and if we grasp the basis upon which the ulcer and its areola are placed, we find that it is swollen or tumid. There is never, when in its natural state, much sensibility. The ulcer may increase more or less according to circumstances; but, if not interfered with, the ulceration will generally stop, and the process of reparation commence before it has attained the size of a shilling.

The approach of the restorative process is denoted by an increased tumidity and rising of the margin of the areola, and by a number of points, of a dull red colour, springing up through the yellowish white surface of the central portion of the bottom of the ulcer. The extent of the areola now diminishes, while the base becomes gradually more full, and the tumidity of the margin of the areola may increase, so as to form a swollen ring round the edge of the ulcer.

The process of granulation usually commences sooner and is more rapid in the centre of the ulcer than at the circumference, and ulceration may continue at the edge and margin, while granulations are forming in the middle. When the granulating stage is at its height, the whole seems full and tumid, and the surface, which, as well as the border, is often considerably raised above the surrounding skin, preserves, if not influenced by treatment, a whitish colour broken up more or less by reddish protruding granulations; the surrounding areola presents at this period a dingy, or livid brown colour.

The process of cicatrization is first denoted by the diseased part seeming less swollen, but chiefly as the granulations shrink, by the edge of the ulcer and corresponding part of the margin of the areola falling inwards, so as to assume the appearance of a ring at the circumference of the ulcer. The outer portion of this ring, as the process of cicatrization advances, becomes somewhat of a callous white colour, and then sends off from its inner redder edge the new cuticle. As these changes continue the ulcer shrinks, and in a few days is found to be entirely covered with cuticle. The surface of the former sore is now greatly contracted, the rounded cutaneous margin still slightly discernible, the newly-formed cuticle furfurates for a time, and the part remains for a long period somewhat livid, and the skin covering it thicker than natural. At length these appearances pass away, but the line which separates the newly-formed integument from the surrounding surface continues, for the most part, ever after accurately defined.

We bear willing testimony to the fidelity with which Mr. Wallace has described this, a not uncommon sore, and we would recommend our readers to study his description carefully. We are inclined to doubt whether, in the majority of cases, the ulcer penetrates through the whole thickness of the skin. When ulceration extends into the subcutaneous adipose or cellular tissue, the granulations are large; in this sore they are always, so far as we have seen them, small. The cicatrix too is not usually deep enough for a sore which has gone through the cutis.

If secondary symptoms are likely to follow the cicatrization, the seat of the ulcer may remain tumid or hard, and may re-ulcerate and heal more than once, before the appearance of constitutional disease; or, when secondary symptoms occur, the original seat of the primary disease will often attract attention from its becoming uneasy or painful. We may here take an opportunity of making a remark which will apply to many primary sores. When a patient applies to a surgeon on account of secondary symptoms, or when a sore has been healed with a small quantity of mercury or without any, it is always right, indeed necessary, to examine the cicatrix. If its colour is unhealthy—if now and then a scab forms upon it, is separated, and then replaced by another scab, and, above all, if much induration remains, it is à priori evidence that the poison is not eradicated, and when assisted by other circumstances, should guide the surgeon in his after-treatment.

Mr. Wallace is not confident of the period that may elapse between the application of the poison and the appearance of "primary syphilis," but he thinks that, on the whole, we may fix it as usually between the third and the seventh day, though it may be either earlier or later.

It is equally difficult to ascertain the time which would be occupied by each stage, if the disease were not interfered with or interrupted. On an average of a number of cases produced by *artificial inoculation*, the phlogosis or redness commences on the second day, the stage of ulceration occupied seven days, that of granulation ten days, and cicatrization six days, making the whole period, from the inoculation to the healing, twenty-five days. Mr. W. thinks, from the observation of ordinary cases, that the periods just mentioned may be taken as a general average, the period of the origin or the commencement of the disease alone excepted.

Having described the symptoms produced by "primary syphilis" on the skin of the penis, Mr. Wallace proceeds to delineate the varieties which it exhibits on other parts.

B. *Primary Syphilis on the Thigh and Scrotum, and at the Orifice of the Prepuce.*

It often occurs in careless persons on the thigh or lower part of the abdomen. Its characters are similar to those already described during the stage of ulceration. But, during the actions of reparation, the base is less tumid, the cutaneous margin less elevated, and the granulating surface of the ulcer seldom rises above the level of the edge. When the disease occurs on the thigh, the cuticle, during cicatrization, not only proceeds from the cutaneous margin, but also frequently commences, without any regular law, on the surface of the granulations, which often present a remarkably tubercular, or an irregular and rounded appearance.

The rugous state of the scrotum favouring cicatrization, the cicatrix is always small, whatever the size of the sore may have been; and to this, perhaps, is owing the remarkable elevation of the surface and border, and serpentine appearance of the latter, often presented by scrotal ulcers during the granulating stage. During the ulcerative stage, the characters are precisely similar to those of the sore on the skin of the penis, save that the surface or bottom of the ulcer is more concave or cup-shaped.

At the orifice of the prepuce. It commences here earlier than on the common skin, and it often presents at its origin a well-formed pustule, with a tumid base. There is generally a plurality of ulcers; they are sometimes of a linear form, and when many such exist, they have often the appearance of radii. Even when round or circular, there are generally one or more fissures traversing their surfaces, corresponding to the folds of the orifice, and giving a chapped appearance to the part. The rim or border is very often so much raised above the surrounding skin, as to form a remarkable ring, and these ulcers are generally remarkable for the tumidity of their base. Lastly, there is a great disposition to contraction during the healing of these ulcers, and from this phymosis almost uniformly results at the period of cicatrization, if not before.

C. *Primary Syphilis on the internal Surface of the Prepuce, at the Corona Glandis and on the Frænum.*

The inner prepuce is a muco-cutaneous structure, as is the covering of the glans. The cuticle in both situations is very fine; and both parts are exposed to much excitement during connexion. Primary syphilis here resembles, in a striking manner, primary syphilis on the external surface of the penis; but there are differences which the dissimilarities of structure and situation are sufficient to account for.

The disease begins early on the inner prepuce, and the pustule is very quickly abraded, so that frequently the first thing noticed is a minute ulcer, of a greyish or yellowish white colour, not larger than a pin's head, resulting from the abrasion of the pustule. From the natural moisture in this situation, there is no scab or incrustation, and the ulcer is generally of a whiter colour than the sore on the common skin. We commonly have a plurality of ulcers here, as at the orifice of the prepuce; but, probably because they are protected, they neither extend so much, nor are so long in healing, as sores on the external surfaces.

This, however, is not the case when the ulcer occurs on the part of the inner prepuce corresponding to the corona glandis, nor when it appears in the fossa and the side of the frænum. In the former situation, the sore is generally much excavated, and often attended by much surrounding induration. In the latter, the ulcer very generally destroys the frænum, having first perforated it from one side to the other. Both often cause a more or less troublesome form of phymosis.

When uninflamed, this is not accompanied by so decided an areola as the sore on the outer integument. The areola must not be confounded with a narrowed margin, which, before and during the stage of cicatrization, here as elsewhere, surrounds the edge of the ulcer.

"Lastly, the greater thinness of the lining membrane of the prepuce, together with the limitation to the process of destruction, and the promotion of the process of restoration, which, as above stated, seem to arise from the protection of the primary syphilitic ulcer in this situation, explain satisfactorily the following additional peculiarities: 1. The absence of that serrated or nibbled margin, which frequently occurs in a very marked degree on the exterior of the prepuce. 2. The deficient excavation of almost all ulcers on the inner surface of the prepuce. 3. The lesser degree of elevation of the border and surface, during the process of reparation; for this stage is often to be known in this situation only by a gentle rising of the surface, surrounded by the narrow red margin above noticed.

It would thus appear, that when primary syphilis occurs on the internal surface of the prepuce, it generally differs from the same disease on the common skin of the penis in the following particulars,—by appearing sooner after infection; by the earlier abrasion or rupture of the incipient pustule, and the consequent formation of a minute ulcer; by the greater whiteness of the surface of the ulcer; by the absence of incrustation; by its being seldom solitary; by its smaller size; by its imperfectly formed areola; by the lesser degree of elevation of its border and surface; and by the absence of the serrated edge. It also appears, that all these peculiarities may be accounted for by peculiarity of situation and structure." 77.

D. *Primary Syphilis on the Glans, and at the Orifice of the Urethra.*

The cuticular lining of the glans is much more closely attached to it than is the cuticle of the prepuce to the subjacent parts. The primary syphilis on the glans differs from that on the inside of the prepuce in these respects; —the ulcer on the glans, while small, is in proportion to its size more excavated; but it is not in general so painful or so sensitive as on the inner surface of the prepuce; nor is there, when this disease occurs on the glans, such effusion, and consequent tumefaction, of the subjacent and surrounding parts, as when it occurs on the prepuce.

It appears to be a property of the glans, when ulcerated, not to fungate to the same extent as many other tissues. During the reparative stage of primary syphilis there are not, in general, granulations formed to fill up the cavity. This stage is for the most part solely denoted by the surface of the ulcer appearing redder, or else covered by lymph, and by an increase of the redness of the cutaneous margin, which becomes depressed or oblique towards the surface of the ulcer; while the surface of the glans itself appears, on the outside of this narrow red margin, paler than natural, or somewhat of a pearl-colour. Probably this indisposition to granulation accounts for the depth and permanency of all cicatrices formed on the glans.

The glans is not so frequently attacked by the disease, nor that so soon after exposure to infection as the prepuce. The latter seldom escapes when the glans is affected, whereas the reverse is often the case. When an ulcer occurs at the corona or angle between the glans and prepuce, it generally makes greater progress in the direction of the prepuce than of the glans.

*Primary Syphilis at the Orifice of the Urethra.* This often occurs, and extends somewhat into the passage. Its general characters are the same as on the exterior of the glans. There is commonly considerable pain during the discharge of urine, and generally cicatrization is followed by a contraction of the aperture.

Cicatrization, when it has once commenced, sometimes advances rapidly, because the new cuticle may proceed at one and the same time from the lining membrane of the urethra, and from the glans at the circumference of the ulceration. When the ulcer of the orifice commences within the urethra, and from that extends over the glans, the healing process may begin in the centre, originating in the lining membrane of the urethra, before the process of ulceration has ended at the circumference,—thus giving to the ulcer an herpetic character of healing in one part, while it spreads in another.

E. *Primary Syphilis at the Edge of the Eyelid, &c.*

This may occur occasionally. It commences by a pustule having the appearance of an hordeolum. During the stage of ulceration, the lid seems as if a piece of it had been removed by a knife, and as if the wound thus caused was sharp and defined at the edge. When the granulating stage has advanced, the surface of the ulcer presents a fungous and yellowish white appearance, precisely like the same form of ulcer in the same stage on many other parts of the body; but as soon as cicatrization commences, this fungous growth shrinks; and when the part has healed, there appears a permanent loss of a portion of the eyelid.

Mr. Wallace observes that primary syphilis may appear in other parts, but it rarely does so, and the general description may suffice. Were we to trust our own experience, we should say that the sore on the lip and on the tongue was more common than that on the eye-lid, and equally, if not more deserving of special notice.

To complete the view of primary syphilis, Mr. Wallace enumerates some varieties.

F. *Varieties in the Origin, Number, Form, Size, Crusts, and Colour of the Ulcers of Primary Syphilis.*

1. Primary syphilis generally commences by a pustule on a muco-cutaneous surface or on the common skin, but more decidedly so in the latter than in the former situation, because the cuticle is firmer. Where there is no cuticle there can be no pustule, and where the cuticle has been removed by ulceration, excoriation, abrasion, wound, or laceration, a syphilitic ulcer may be formed, independently of a pustule.

2. Primary syphilis is not always solitary, because the poison applied to several parts may infect each.

"I have ascertained experimentally, that the secretion from a primary syphilitic ulcer can produce, when applied to a sound surface of the same individual, a similar ulcer; therefore we are authorized to conclude, that when a plurality of these ulcers occur at one time in the same person, they may have been caused not only by the application of the original poison, as has been already said, but also by the secretions from a syphilitic ulcer existing at the same time in another part of the same individual; and it is remarkable, that those ulcers which appear last run their course, like the vesicles of cow-pock under similar circumstances, much more rapidly than those which appear first." 82.

This is familiar to all who see much of venereal patients, independently of experiments. A sore on the inner prepuce will produce another precisely similar in every respect on the part of the glans with which it is in contact; a sore on the dorsum of the penis will produce a sore on the pubes to which the penis is opposed when supported by a handkerchief. In women nothing is more common than to witness a series of pustular sores on the edge of one labium, corresponding exactly with a similar series on the other. Mr. Wallace has not alluded to these facts. We see such daily. On the whole, it is a vulgar fallacy to suppose that the number of sores makes against their genuine syphilitic character. We have under our care at the present moment a patient who has eight or ten on the penis.

3. The figure of the sore may vary. It is true that it is generally disposed to be circular, but where these folds in the integument, as at the outer prepuce, it may be linear, or from the union of two or more sores it may assume an indefinite variety of figures. We very often see a sort of hour-glass sore on prepuce and glans at the frænum, or at the corona, from the junction of two at one edge.

4. The size may vary without much variation of character.

5. The incrustations vary. They are sometimes soft and spongy, and at other times firm and hard. They sometimes fall off quickly, and at other times adhere for a long period. Those which fall off quickly are in general of a light colour, and those which adhere for a long period are more dark. Sometimes they are thick and tuberculated, and at other times, being thin and transparent, the diseased surface may be almost discerned underneath them. This, however, seldom happens, unless when the crust has been recently detached, and the new one has not acquired much thickness. When crusts are firm and adherent, they indicate a slow disease; but when they are soft and spongy, and fall off quickly, the disease is more rapid. When crusts are very firm and adherent, they will sometimes not fall until the surface be healed underneath; and when they adhere until the granulating stage is advanced, they are often surrounded by a tumid ring at their base, owing to that elevation of the border which characterises the stage of granulation. These adherent crusts may form long and projecting scabs of a pyramidal shape, or they may be composed of flat concentric circles. Crusts being the result of desiccation of discharge, cannot of course take place when desiccation is prevented.

6. The colour, though a good general guide, is not a certain one. It is mostly a slate or ash-coloured white, a yellowish white, or a dirty white. But the colour is not always constant. We would say that it is much affected by cleanliness, dressings, treatment, or state of the patient's system, and diet. The most usual colour is certainly a yellowish white.

G. *Observations on the Diagnosis of Primary Syphilis.*

This is rather a recapitulation of preceding observations, and adds nothing we think to their clearness. There are two general remarks which we think so true as to merit attention. The first is that there is no one diagnostic mark of a syphilitic ulcer, nor any combination of symptoms decisive of the point. The only way towards arriving at correct conclusions is to weigh all the circumstances, the appearances, the history. The man of observation and experience will generally be right. The second remark is this, that minute descriptions, however prolix they may appear, are absolutely necessary, as are minute observations, in a disease like this. The routinist is a sorry animal, and Mr. Wallace's picture of him is a true one.

"Although a routine practitioner may, by long-continued experience, and after the expense of many blunders, acquire a sort of knowledge, which will enable him to judge, without knowing why, that the disease under his observation is better suited to one system of treatment than to another, he will never be able to communicate to others the principles upon which he acts; nor will he ever be able to point out to his own mind those circumstances by which his judgment is directed, and consequently he will often be in danger of falling deeply into error, before his error is discovered. In short, he may, after long experience, act in general correctly, although empirically. But, whenever we can act upon principle, empiricism is unpardonable; for although it may sometimes succeed, it is much more frequently a source of evil than of good." 89.

### Treatment of Primary Syphilis.

Mr. Wallace indulges in some preliminary observations, the gist of which is that we should not treat a case of syphilis blindly, but should pay attention to the health of the patient and the state of the parts. Nothing can be more judicious. Because a patient has syphilis we are not on that account to let him become pale, emaciated, and debilitated in every way, nor, on the other hand, are we to allow the parts to become inflamed, or irritable, or indolent, and not adopt the ordinary means for such conditions. The great practical rule is this—maintain the general health, and treat the local actions on general principles. The means of doing so must be left to the discretion and the experience of the individual practitioner, as no general rule can be given. We would simply make this remark—the patient should not live low, and if he has been accustomed to any particular stimulus, either that or an adequate substitute should be allowed him.

A. *Treatment of Primary Syphilis during the first and second Stages.*

Our readers will remember that these are the stages of destruction, the third and fourth being those of granulation and cicatrization, or of repair. The disease is seldom seen in its very first stage, or that previous to ulceration. Mr. Wallace recommends that, if seen, the part should be cut out, but the patient should still be treated constitutionally, as if the disease had been more advanced. We confess that we doubt the propriety of this advice. Either the excision is effectual or it is not. If it is, we require no constitutional treatment; if it is not, why resort to it? The character of the sore, when it forms, and its progress, are of great assistance to the surgeon, in enabling him to judge of the propriety of his constitutional treatment. If we excise the part we lose this index without any equivalent advantage.

Patients generally apply in the stage of ulceration. In that stage Mr. W. immediately applies the nitrate of silver in such a manner as to destroy the diseased surface. In a healthy person this, he says, will stop the process of ulceration, and by preventing the necessity for granulation, lead directly to cicatrization. But if the process of reparation is already commenced "nothing can be gained, but much injury may be done by an escharotic application, for at that period all the actions are tending to cicatrization and the destruction of the newly-formed granulations must have the effect of retarding this process." Now this is a point of practice, a matter of fact, in which we feel compelled to differ, toto cœlo, from Mr. Wallace. We are not satisfied of the truth of either of his positions. We may mention a case in reference to the first. A patient had two sores decidedly syphilitic on the body of the penis. They were in the stage of ulceration. We dressed one with the black-wash and treated the other with caustic, the patient taking at this time the blue-pill. They both improved pari passu, the one touched with caustic rather lagging behind its fellow, than out-stripping it. We have seen much inflammation and very troublesome consequences from the application of caustic in this stage. On the other hand we have no hesitation whatever in saying that caustic in our hands has expedited, or most unequivocally appeared to expedite the healing of the sore, when applied in the stage of granulation. We might bring forward many cases in proof of this assertion, did our limits permit us to indulge much in critical observations. But we may remark that analogy is altogether against Mr. Wallace. We would ask if the nitrate of silver is found to retard the cure of ordinary ulcers in the stages of granulation and cicatrization? Any hospital dresser would reply in the negative.

On the whole, then, we have arrived at a conclusion opposed to that of Mr. Wallace. In the stage of ulceration we would rather use simple applications, as the black-wash, and see the local effect of our constitutional treatment, whilst in that of granulation we would employ the nitrate of silver. And this is not so much from theory as from the result of the cases we have witnessed and have treated.

The following remarks on the ulceration of or near the frænum, perfectly agree with our own experience, and are highly deserving of attention. We have found the nitrate of silver in these cases quite as effectual as Mr. Wallace reports it. In one case we tried the compound tincture of benzoin, but it failed.

"In treating primary syphilis situated near or on the frænum of the prepuce, I have, on many occasions, demonstrated to the pupils at the hospital the remarkable influence of the nitrate of silver in stopping the ulcerative process of this disease. The tendency which chancre has, when it occurs in the fossa between the corona glandis and the frænum, to perforate this fold is notorious; and so universal is the belief, that when it has been perforated, the ulcer cannot be healed until the whole of this membrane be destroyed, that it is laid down in books as a rule of practice, to divide the remainder of it with a bistoury, as soon as the perforation is observed. Now I affirm that, in nineteen cases out of twenty, if the patient applies before the ulcer has perforated the frænum, its perforation may be prevented, by employing the caustic in the manner here recommended; and I still further affirm, that if he has not applied until after its perforation, we may, if we think it right, still save the remaining portion, by cauterizing with the nitrate of silver the sides of the opening. If the hole be sufficiently large, the caustic should be run through the perforation, so as destroy completely the diseased surface. In this manner I have, on some occasions, but simply with the view of demonstrating the power of the caustic, saved the residue of the frænum, when it was scarcely thicker than a hog's bristle, and separated from the penis by a hole sufficiently large to allow a pea to pass. A delicate string of this kind is, however, in general ruptured by the first erection; but, at other times, the hole in the frænum has been so minute, that a hair only could be passed through it, and then this membrane continued to be as useful as before the perforation; which perforation however never filled up." 96.

Our author gives many minute directions on the manner of applying the caustic. For these we must refer to the work itself. One passage, however, we will quote, because it contains what approaches to gasconade.

"The fear of inflammation is totally groundless; the dread of bubo, we shall hereafter see, is if possible still more groundless; and the inconvenience presumed to arise from the want of a criterion to regulate the constitutional treatment after the healing of the ulcer, is a phantom of the imagination, suited only to amuse the patient, and to reconcile him to a system of practice most unnecessarily cruel, because most unnecessarily tedious,—in fact, we have many more satisfactory guides to regulate our course." 99.

We do not hesitate to say that there is here a great deal of exaggeration. The student would suppose that "the unnecessarily cruel" treatment of sore, that is the non-application of caustic in the early stage, must lead to a very tedious affair. We most positively assert our conviction that, under judicious management, this is not the case; that sores well treated, locally and constitutionally, heal without caustic in the ulcerative stage, as well as, or better than, with it, and that the cruelty and the tediousness are phantoms of the imagination of the ingenious author. We also venture to contend in the face of Mr. Wallace's somewhat positive denunciation, that the disposition to healing of the sore under simple applications and general treatment, *is* a criterion of some importance, and one that a good practical surgeon is always well pleased to have. In conclusion, we would hint that Mr. Wallace himself is somewhat too caustic in his remarks as well as in his treatment. It is hardly decent to accuse all who do not use caustic as freely as himself, of "founding their opposition on prejudice or timidity, both of which generally spring from ignorance." This is unhandsome. We suppose we must be content to be considered prejudiced, timid, or ignorant, for we cannot agree with Mr. W. in his unbounded love of caustic. As Boswell says, what will fill a pint pot won't fill a quart—the evidence against caustic has filled us, but not Mr. Wallace—and we must therefore be looked on as the pint pot.

B. *Treatment of Primary Syphilis during the Third and Fourth Stages.*

We now arrive at the consideration of the mercurial treatment, a consideration as vast and as important as any in the circle of medicine. Mr. Wallace observes very justly that the world is divided between two opposite errors—one, that mercury is indispensable for the venereal disease—the other, that it is not required for it. Men are see-sawing between these notions, and the practice at the present moment is a strange jumble of their discordant elements. We subjoin the following remarks, as very exactly descriptive of the present indecision.

"No doubt very great mischief has resulted in the practice of routine or unreflecting practitioners, from the long-formed and deeply-rooted opinion, that mercury was an unerring specific against every form of venereal disease. For this opinion led to the supposition, that, whatever effects the venereal poison produced, these effects were controllable by the action of the specific; the modifying or opposing influence of collateral circumstances was not taken into account; and to remove the effects of the poison, it was deemed necessary only to proportion the quantity of the remedy to the obstinacy of the disease.

It would also seem that the belief in the all-powerful influence of mercury over the progress of venereal diseases had naturally led to the conclusion, that these diseases could not be controlled, unless by the influence of this remedy. From these conjoined opinions—the belief in the paramount efficacy of mercury, and the belief that this remedy was indispensable—the most direful consequences resulted: consequences vastly more destructive than those produced by the uncontrolled influence of the venereal poison.

Although it seems to have been known to many of our predecessors that the foregoing opinions were erroneous, their writings on the subject did not obtain that attention which they so highly deserved; and it was left to the researches made in our own times to demonstrate, beyond doubt, that mercury does not control, under all circumstances, those states which are produced by the venereal poison; and that the effects of this poison are not necessarily progressive unless controlled by mercury. These important discoveries—and important discoveries they may well be called—must, if used with discretion, contribute in a remarkable degree to an improved mode of treating the venereal disease; and in the future parts of this work, their value will appear in full light. But on this occasion, as on almost all others, we run from one extreme to another; and the erroneous opinion that mercury necessarily controlled the effects of the syphilitic poison, and that the diseases produced by the influence of this poison were necessarily progressive unless controlled by mercury, has been in many cases superseded by the equally incorrect opinion, that this medicine does not exercise any specific influence over venereal diseases; and that in their treatment more injury than advantage always results from its action." 104.

The great error is, that men have expected too much from mercury on the one hand, and, upon the other, that they have not investigated its peculiar effects, and the manner in which it may modify venereal symptoms.

Let us now look a little more narrowly at the arguments used by the mercurialists and the non-mercurialists.

The chief objections urged against the use of mercury by the non-mercurialists are three;—1, that mercury is not a specific, some forms of the disease resisting it; 2, that the venereal disease is curable without mercury, and that as easily as with it; 3, that mercury is often productive of effects as injurious as the disease for which it is given. Two of the arguments, in short, are directed against the use of mercury, one against its abuse. Let us look at them seriatim.

And first, that mercury is not a specific. It appears to us that very erroneous ideas are entertained on this head. There is no such thing as a specific, in the extreme sense of the word. Bark will not always cure ague, brimstone will not always cure itch. But in the great majority of cases these remedies, properly timed and judiciously employed, will cure the diseases to which we have alluded, and there are no other remedies so effectual nor so certain. We maintain that mercury has as good a title to be considered a specific in syphilis as cinchona in intermittent fever, or sulphur in scabies. We need not say that, if this be true, it cannot be a valueless remedy.

The second argument employed by the non-mercurialists is, the curability of syphilis without mercury. This is certainly an important one, and if founded on general experience it would prove a conclusive one. But is it founded on general experience? We will first introduce Mr. Wallace's opinions and reasoning on this subject.

" It is affirmed, that primary venereal diseases of every form or kind may be cured without mercury. Now, although I am not prepared to go so far as to admit the possibility of curing primary syphilis on every occasion without mercury, I am prepared to say that a vast number of cases of this disease may be so cured. But does this, or even the universal curability of syphilis without mercury, form a positive objection to mercurial treatment? This question cannot be answered without first determining, whether more advantage may be obtained from this, than from any other mode of treatment; and secondly, whether any positive or counterbalancing injury results from the proper employment of mercury in the treatment of primary syphilis; for we must not argue against an appropriate use of a remedy by adducing consequences which may arise from its improper or injudicious administration.

In answer to the first question I reply, that it must be admitted, even by the opponents to mercurial treatment, that in a large number of cases of primary sores, the length of time required for their cure is greatly diminished by the employment of mercury, and also that this medicine has very considerable influence in preventing secondary symptoms or contamination of the system; while in answer to the second question, I must content myself for the present with affirming, that I have never observed any injury whatever to result to the constitution of the patient, from that employment of mercury in the treatment of primary syphilis, which the reader will find recommended in the future pages of this work.

Now when syphilis can be cured more rapidly with mercury than without it, and when no inconvenience can result from a properly directed administration of this medicine, have we not the same reasons for using it in primary syphilis, as we have for using any other remedy in any other disease? We do not in general employ medicines, because of a conviction that the diseases for which they are employed would be incurable without them, but because we know from experience that the cure of disease is often rendered more rapid by a judicious interference of art. Precisely the same principles should guide us in the treatment of primary syphilis.

Suppose that a patient, possessed of ordinary powers of forming a correct judgment, applied to a surgeon, on account of primary syphilis, and that the surgeon candidly assured him, that, although the disease under which he laboured might be cured without mercury, yet that its cure would most probably be hastened by the employment of this medicine; and suppose that the surgeon further informed him, that great mischief might result from the improper or incautious use of mercury, and then left the patient at liberty to decide which mode of treatment he would prefer; I can, from experience, say, that he would indubitably prefer the treatment by mercury.

Let it be admitted that secondary symptoms will not occur oftener, on an average, than in one case out of twenty, or in five in the hundred, which have been left entirely to Nature: this being the lowest average which has been made on this subject;—and let it be further admitted, that of these five cases, which would be followed by secondary symptoms if left to Nature, no more than one would be followed by such symptoms if the primary were treated judiciously by other means than mercury: this would leave on the whole only one case in the hundred to be followed by secondary symptoms, when mercury was not employed. Now put the question to the patient in even this extreme way, or let him be told that there is only one chance out of the hundred that secondary symptoms will occur, although he may not use mercury, but that the chance would be diminished if mercury were employed; and I have no doubt, that, under almost all circumstances, this treatment would be preferred by him; but particularly so if he be informed of the fact, that the same treatment, which will be most likely to prevent secondary symptoms, will also promote the cure of the primary, and that the same care or caution in his mode of living, which is in general necessary for the cure of the primary sores without mercury, will be all that is necessary in conducting an appropriate mercurial course.

On the whole, while I admit the important results, which have sprung from modern inquiries respecting the venereal disease and the action of mercury, and feel sincerely grateful for the addition thereby made to our knowledge, and particularly as to the determination of the question of the general curability of venereal diseases without mercury, I must express my conviction, that much mischief has arisen from the general cry raised against this medicine, and from the vacillating and unsteady practice, to which this injudicious clamour has led. These modern prejudices are now however ceasing—not gradually but rapidly; and I have no doubt that ere long a middle course of practice will be universally adopted; and that the evils of the old mercurial, and of the more modern anti-mercurial practice will be equally avoided, and a rational system of treating the venereal disease adopted in their place,—founded upon a knowledge of the facts, that mercury, if properly administered, is in a great number of cases highly efficacious in controlling the venereal disease, or that form of morbid action, which is produced by the influence of the venereal poison; that this disease may however be in general cured, if necessary, without mercury;—and that, on some rare occasion, this remedy, in place of curing syphilis will aggravate all its symptoms. In short, with these facts, which have been satisfactorily ascertained by modern researches, and which are now placed before our eyes, we shall no longer be in danger of employing mercury, when more mischief than good may result from its employment; nor of persevering in its use, when it can no longer serve any good purpose, but may produce the most injurious consequences. We have however much still to learn; and it is the duty of every practitioner, who possesses opportunities, to take advantage of them, and endeavour to arrive at fixed rules of conduct in respect to many points as yet unsettled." 109.

The chief facts on which the argument against the necessity for mercury has been founded, are those contained in the Army Medical Reports. They are certainly staggering, and we must confess that, taken *per se*, they would naturally exercise a powerful influence on the mind of any candid enquirer. But even in these reports there are considerable discrepancies—the non-mercurial practice appearing more successful in some instances than in others; and when we consider the perfect control which can be exercised over soldiers in hospital, and the regularity in diet and in discipline which can be enforced, we might suppose that the results of any experiment would be most certain and most satisfactory in their persons.

The facts elicited by the army surgeons have made a great impression on the minds of all professional men, and few, if any, have refrained from making experiments on the treatment of sores without mercury. We believe that, if the majority of those connected with institutions where venereal patients are collected, were required to state the results of their experience, it would be in favour of mercury. We venture to say that there is not a surgeon in extensive practice in London who has not abandoned, if he has tried, the non-mercurial treatment. Questions of this sort are generally best determined by the common experience of mankind, and we entertain no doubt whatever that this is on the side of mercury.

It would be difficult to bring forward numerical statements in opposition to those in the army reports, and any such attempt would be out of place here. But we may state broadly, that all that we have seen is opposed to the tenor of those reports. We do not say that syphilis cannot be cured without mercury, but we do say that all we have seen has compelled us to this conclusion, that syphilis is cured more speedily, more easily, and more effectually with it than without it. We have under our care, at the present moment, four cases of relapse after the non-exhibition, or the inefficient exhibition of mercury. One patient had sloughing sore on the penis, and sloughy throat. He was cured by sarsaparilla and general tonics. He has lately returned to us with extensive ulceration of the throat and pharynx, an interval of some months having elapsed between the first and the secondary affection. The second patient had also a foul sore on the penis, and sloughing ulceration of the throat, which were cured by sarsaparilla, &c. He returned with broken health, sallow complexion, pains in the limbs, and ulceration in the throat. The third patient had a sloughy sore on the penis, which was cured by tonics, and a very small quantity of mercury, continued only for a week or ten days. He has returned with pains in the limbs, emaciation, sallowness, and all the precursory symptoms of some secondary affection, sore throat, or eruption. The fourth patient had the tubercular eruption. He was treated inefficiently by the oxymuriate, under which the eruption nearly passed away. He has returned emaciated, and covered with clustered tubercles, the former having been insulated. He is rapidly improving under the use of the mercurial ointment.

We have said that it would be difficult, and out of place in a review, to meet the argument we are now considering by specific details. We have merely adduced the preceding cases as happening to be under our observation at the present moment, and we put it to any practical man, whether such occurring frequently cannot fail to make a great impression on such as see them. On the one hand the experienced surgeon reads documents calculated in an eminent degree to shake his belief in the necessity for mercury; on the other, his daily observation serves to confirm it. What is he to do? That happens which might, à priori, be supposed to happen. The surgeon with little experience is confounded and dismayed, and flounders on either blindly giving or blindly withholding mercury. The man of greater opportunities disregards what he reads for what he sees, and forms for himself a practical code of treatment, a code into which we feel certain that mercury enters pretty largely. Such we believe to be the state of the case at the present moment. Experienced surgeons are, upon the whole, moderate mercurialists; surgeons with little experience either do not use mercury at all, or abuse it. This is a condition which is far from satisfactory, and it is on this account that we wish to bring the subject of the venereal disease and its treatment prominently and frequently before our readers.

The third argument against mercury hinges on its abuse. To this we need only reply, that if a powerful remedy be abused, the best method of correcting that abuse is not to interdict its use.

After all, the practical conclusion to which we must come is this—that surgeons should carefully observe the character and watch the progress of venereal sores and symptoms, that they should also carefully mark the effects of mercury, and, having done this, they will be best able to determine the conditions that are benefitted by mercury and those that are aggravated by it; when mercury should be given and when it should be withheld.

We return to our analysis.

Mr. Wallace properly observes, that the precise mode in which mercury acts is a mystery which passes our powers of observation. It is one of those ultimate facts which always baffle inquiry. The legitimate subjects of observation and investigation are the phenomena displayed during its employment. Mr. Wallace seems to consider mercury a stimulant. What his precise notion of a stimulant may be we cannot say, but certainly mercury is no stimulant in the sense in which alcohol or bark are stimulants. If an individual take wine, an acknowledged stimulant, for a certain length of time, he will under ordinary circumstances become more robust, more plethoric, more liable to the diseases of repletion than before. Just the contrary is the case in respect to mercury. The individual who takes it becomes pale, emaciated, irritable, weak, and if a certain limit be passed, he dies of a disease the very reverse of repletion. Nay, we assert most positively that, stimulants are the antidotes to mercury—that a patient going through a course of mercury should live comparatively well, that if accustomed to porter or wine he should have porter or wine, in short, that to prevent the injurious consequences of mercury he should be *stimulated*. Do we find that the effects of opium are diminished by another narcotic—that the debilitating consequences of one medicine are diminished by another which debilitates also? We apprehend not, and we are convinced that if there is one fallacy more gross and more pernicious than another, it is that of considering mercury a stimulant. It is an abuse of terms, an egregious scholastic delusion, that we know not how to condemn sufficiently. We say to the profession, do not think mercury a stimulant, but be content to watch its effects in health, its power of controlling action, and the kind of action that it controls in disease, and then be guided by an acquaintance with those effects, in exhibiting or refraining from it. It is the insane desire of writers on the Materia Medica to nosologize, that has led them to put medicines in rank and file, when the fact is, that the same medicine under different conditions of the recipient has different or opposite effects, and no two which are placed together have really a common action.

Mr. Wallace observes that great mischief frequently results from the topical application of mercury, or other stimulants, during the stage of ulceration or destruction, the most appalling cases which he has witnessed having been caused by the injudicious application of the red precipitate, or other irritating substances. This agrees in a great measure with what we have seen, but we may be permitted to remark, that it does not agree with Mr. Wallace's strenuous recommendation of caustic in the early stage of sore. We have seen the black wash ("a topical application of mercury") agree well with the sore throughout, and really we feel no objection to using it throughout. But the application of caustic in the early stage has, in our observation, been followed, in one or two instances, by those very injurious effects that Mr. Wallace attributes to stimulating or irritating applications. We put it to any person whether nitrate of silver is not a greater stimulant or irritant than the black-wash? We think Mr. Wallace rather biassed by his theory on the destructive and reparative stages, in condemning the application of all stimulants prior to reparation. The great rule on which we should insist, is not the observation of the slight barrier between these stages, but of the presence or absence of inflammation. If the sore is angry or inflamed, it is better to employ poultices or tepid emollient applications until the inflammatory action has subsided. But this is merely an application of the general principles of treatment of sores, venereal and not venereal.

Mr. Wallace also condemns the internal exhibition of mercury during the ulcerative stage. Here, again, we feel a difficulty in agreeing or differing with him. It appears to us, that it is not the ulcerative stage, per se, that forbids the exhibition of mercury, but a state of system unfavourable to its employment. If a patient applies with an anxious or unhealthy appearance, a white or loaded tongue, a frequent pulse, a warm skin, and, as usually happens under these circumstances, an inflamed appearance of the sore, the employment of mercury would be most injudicious, and would probably give rise to sloughing or to phagedæna. If, on the contrary, the patient appears with all the symptoms of debility, emaciated, pallid, with a feeble pulse, the exhibition of mercury would be equally improper. It may be said, as Mr. Wallace seems to say, that mercury is pernicious in the former state, because it is a stimulant, and the patient is already in a state of excitement; but then it ought to agree in the latter state, which it certainly does not. A patient labouring under acute phrenitis is in a condition of extreme excitement, yet mercury is given, and is highly beneficial. The fact appears to be, that the diseases in which the most acute inflammation, the most extreme excitement prevails, are those in which mercury effects its greatest miracles. It is not because the patient with an inflamed sore is in a state of excitement, that mercury is not indicated, but it is because a certain state of system, one in which the secretions are tolerably healthy, the functions tolerably well performed, the vascular action neither above nor below par, is the best adapted for a mercurial course. To this condition the patient must be brought—if he have pyrexia and depraved secretions, by diaphoretics, and quiet, and purgatives, into which mercury may freely enter—if he be debilitated, by generous diet, sarsaparilla, tonics or stimulants, according to the circumstances of the case. Such seems to us to be the practical rule in the administration of mercury, such we have acted and act upon, and we have never had reason to regret our having done so. To return to Mr. Wallace—

"It can scarcely be necessary to observe here, that the rule of abstaining from mercury during the stage of ulceration, as well as the other directions given in this chapter, are applicable only to the regular form of primary syphilis; for, if the actions of reparation be so very slow in commencing as to lead us to fear contamination of the system; or if the ulcerative action be advancing in any case with such rapidity as to endanger the loss of parts of importance, &c. &c. we must act on a different principle, as will be pointed out hereafter. What I have said hitherto, applies, therefore, solely to those cases of primary syphilis, that are not complicated; or, in other words, that are not attended by any of those diseased states or actions, which, whether depending on constitution, mode of living, or previous treatment, &c. produce such varieties of disease, as deviate more or less from that above described, and hence require corresponding peculiarities of treatment." 111.

The topical mercurial applications enumerated by Mr. W. are, in succession from the least stimulating to the most so,—calomel, calomel in lime-water, mercurial ointment, citrine ointment, muriate of mercury in lime-water, and muriate of mercury in distilled water. The milder application should be used first; it is often advantageous to alternate them, and sometimes to intermit for a day or two all mercurial applications, using in the meantime weak solutions of the nitrate of silver, or the sulphate of copper, or distilled water, or an aqueous solution of opium. These and other stimulating applications should be used according to the condition of the sore. We fully agree with Mr. Wallace in his wish to cure the primary sore as speedily as possible. When once we have made up our mind as to its nature, we may as well accelerate cicatrization by judicious topical applications. We condemned the application of caustic in the first instance, because, though Mr. Wallace denies that he has witnessed unpleasant effects from its employment under such circumstances, we maintain that we have: because it is often very difficult to pronounce at first upon the character of the sore, and caustic mystifies it: and, lastly, because we always desire to see a sore taking on a healing action under the general treatment, the best indication of its propriety. When once we are satisfied of the nature of the sore, and when the general treatment is manifestly agreeing with it, there can be no good reason for allowing it to remain unhealed one day or one hour longer than is necessary. We now arrive at the mode of exhibiting mercury internally. We will give Mr. Wallace's sentiments and mode of practice.

"In dispensary practice, and among the lower ranks of society, the internal administration of mercury, particularly at inclement seasons of the year, can seldom with safety be recommended. In such persons, and under such circumstances, topical applications are of infinite value. In cases of this kind, I generally confine my treatment to them, in conjunction with the internal use of nitrous acid; and by these means, I succeed for the most part in healing the disease with rapidity. Cases treated in this way are also very seldom followed by secondary symptoms. Whether this be owing to the topical application of caustic and mercury, or to the internal employment of the acid, I cannot as yet pretend to say decidedly; but probably both contribute.

The blue pill and the submuriate and muriate of mercury by the stomach, mercurial ointment by friction, and the red sulphuret of mercury by fumigation, —are the mercurials commonly employed by me in treating primary syphilis, with the view of exciting and supporting the necessary degree of mercurial action in the system.

Whatever mercurial preparation be used, it will be always most prudent to combine it with opium and antimony. No harm can result from this practice; and by it, much inconvenience may perhaps be avoided. The combination of antimony with mercury has always appeared to me to render the influence of the latter more manageable, as well as more certain; while the addition of opium diminishes the irritating influence of mercury on the bowels, and subdues the disposition to an irritable state of the general system, or of the local disease.

Calomel combined with opium, and with either antimonial power or tartarized antimony, is a very useful form of medicine. In the greater number of cases, this combination may be used until a sufficient degree of mercurial action has been excited; but it should in general be omitted as soon as the gums or breath testify even the mildest influence of mercury; and a solution of the corrosive muriate substituted for it. By this medicine the mercurial action may be kept up in a sufficient degree, without the risk of exciting a troublesome salivation; and this change of the mercurial preparation will also be as beneficial as the alteration of the topical applications, already recommended upon the same principle. This principle may be carried even further with great advantage; and mercury may be omitted altogether for some days; during which the patient may take the nitrous or any other mineral acid, either with or without sarsaparilla.

The quantity of calomel required will vary in different individuals. I do not in general hesitate to begin in common cases with six grains a day in divided doses, combined with the same quantity of antimonial powder, or with the eighth of a grain of tartarized artimony, and one grain of opium. It is, sometimes, necessary to increase, or even to double the dose of calomel. At other times, it will be prudent to employ smaller doses, but in this case I would prefer to give five grains of the blue pill night and morning, combined with a quarter of a grain of opium, and with tartarized antimony, or antimonial powder, if the stomach will allow either.

When the muriate is administered, two-thirds of a grain of this salt, dissolved in a sufficient quantity of distilled water, may be given, in divided doses, during the twenty-four hours. But should this solution disagree with the stomach or bowels—an event which not unfrequently happens—it must be omitted, and the blue pill combined with opium, &c. given in its stead.

Friction with mercurial ointment, or fumigation with the sulphuret of mercury, will not be necessary, unless there exists a natural delicacy of bowels to the action of mercury: or unless the system is not easily influenced by this medicine, when taken internally. In case it be ascertained from former experience, that the individual is very susceptible of mercurial influence, the muriate in solution, or the blue pill with opium and antimony, should, from the commencement, be employed.

Although I am not prepared to deny altogether the hypothesis of Hunter and Delpech, that a peculiar advantage results from employing mercurial frictions in primary syphilis, in consequence of the mercury being thereby conveyed into the system through the same channel which conveys the virus, I very seldom think of directing them, because of the great objection which patients have to their employment, and because of the obvious inconvenience attendant on them.

A very mild mercurial action must be kept up in the constitution, not only until the ulcer is perfectly healed, but if there exists no cause of objection from adventitious circumstances, until all tumidity or hardness of the base upon which it was placed has been removed; for this is the criterion by which we are principally assured that all diseased action has ceased. It is at the same time to be observed, that now and then cases occur, in which the disease leaves after it a state of callous induration, which is persistent, and cannot be influenced by treatment. A little attention will, however, enable us to distinguish such cases from those in which we should persevere in the use of mercury until all callosity has been removed. It should be also remarked, that the ulcer may heal, and that all induration may be removed sooner than it would be prudent to leave off mercury. In these latter cases, although we must take into consideration the quantity of this medicine which has been used, and the effects which it has had on the constitution, it may be said that the system should be kept under the mild influence of mercury, for ten days or a fortnight, after the ulcer of primary syphilis has cicatrized." 116.

It is an important fact, if strictly correct, that Mr. Wallace's dispensary patients are usually cured without the internal exhibition of mercury. We do not find that the out-patients in our London establishments either bear mercury badly, or are more curable without it than other persons. We have treated many under these circumstances, and have never seen reason to regret its employment, and frequently we have been compelled to resort to it from the supervention of secondary symptoms, or the inadequacy of other modes of treatment.

There is a point on which we cannot altogether agree with Mr. Wallace. He seems to give the preference to calomel among the mercurial preparations, and he observes, that as soon as the gums or breath testify even the mildest influence of mercury, this (the calomel) should be omitted, and a solution of the corrosive sublimate substituted for it. We are fully aware of the advantage of occasionally changing the mercurial preparation, but the circumstances that appear to us to call for the alteration are, a difficulty of affecting the mouth by one form, or, if the mouth is affected, an obvious tardiness of improvement. Under such circumstances, we have seen very marked advantage from substituting one mercurial preparation for another. But we do not so clearly understand why, if one preparation is succeeding, it should be superseded by another, and that the least efficient and the most capricious of all, the oxymuriate. In fact, we like neither the calomel in the first instance, nor the oxymuriate in the last, the former being as much too active and too debilitating as the latter is too uncertain. Mr. Babington has remarked that calomel seems best adapted to those cases in which the tone of the system is high, the patient vigorous, and the disposition towards inflammatory action. Under other circumstances, the milder preparations, the blue-pill, or the mercurial ointment, are preferable to it. We had rather put a patient on a steady course of one or other of these than adopt Mr. Wallace's method.

Whilst we make these remarks we are far from undervaluing Mr. Wallace's caution with regard to the mercurial action on the system. We trust that the days of salivation and erythismus are past, never to return. In the immense majority of cases there is no necessity for doing more than gently affecting the mouth; this is a sufficient test of the mercurial influence, and beyond this we need not carry it. But we feel inclined to think that this can be done as well by one mild, though efficient preparation, as by two or several. There can be very little question, at least there is little with the surgeons of the greatest experience in these matters, that the mercurial ointment is, on the whole, the mildest and the most certain of the forms of mercury. The great disadvantage attending it is its inconvenience, which renders private patients averse to it. Next to the ointment we would rank the blue-pill. Mr. Wallace's combination of opium and antimony with this is good, but we have found the junction of conium generally effectual in preventing any tendency to griping or diarrhœa, and less objectionable, in some respects, than that of opium. The dose may be five grains of the blue-pill and five of the extract of conium, to be taken twice daily. We do not think that Mr. Wallace has sufficiently dwelt on the relative effects of the various forms of mercury. Each is of use under certain circumstances, but their selection must be guided by considerations into which Mr. Wallace might have entered more fully with advantage.

It will be observed that Mr. Wallace lays down two criteria to determine the time during which the mercurial course should be continued—the healing of the sore, and the disappearance of induration in the cicatrix. The remarks on the latter head are extremely just. In order to shew how carefully the state of the cicatrix should be attended to, we will mention one case out of those which are occurring to us almost daily. A gentleman had three sores on the inner prepuce, the appearance and history of which led us to believe that they were decidedly syphilitic. In consequence of some derangement in his general health, we merely prescribed light purgatives. The sores healed in a week or ten days, and a bubo, which existed in the groin, had not increased. A surgeon of some experience now saw the case in consultation, and recommended that no mercury should be given. We were of the contrary opinion, but it was finally determined that the patient should take his chance without the mineral. In a fortnight or three weeks from this time the gentleman again came to us. The cicatrices of the sores were severally indurated, feeling like peas in the skin, the colour of the latter was rather florid, and the bubo was in a state of suppuration. We put the patient on five grains of blue-pill twice daily, and gave him sarsaparilla: the bubo was opened, the skin, which had been extensively discoloured and likely to ulcerate, recovered itself, the cicatrices lost their induration, and, after going through a course of five weeks, the gentleman left town in a state of better health than he had felt at the commencement.

While we cordially agree with Mr. Wallace on the propriety of carefully attending to the condition of the cicatrix, we feel disposed to doubt the safety of his advice on the discontinuance of mercury so soon as ten days or a fortnight after the cicatrization of the sore. Mr. Wallace is speaking of an ulcer which heals early, and, by judicious local applications, the syphilitic sore will often cicatrize in the course of a week. Under such circumstances, we should not consider it prudent to keep up merely a mild influence for ten days or a fortnight afterwards. It appears to us that if we subject a patient to mercury at all, we should not do so by halves. Experience proves but too truly, that a great majority of the cases of secondary symptoms are those in which mercury has been given for only a fortnight or three weeks. In general we would give a course of a month or five weeks, however early the sore might have cicatrized. Under judicious management the patient's health will be little, if at all, the worse for a course of this duration, and if he is rendered more secure we cannot comprehend why it should be curtailed.

Mr. Wallace observes that there is not, in general, a necessity for rigid confinement, and that the patient may follow his usual avocations, if he protect himself from cold and damp. Temperature and climate exercise an undoubted influence over the disease and its treatment. In mild and temperate regions it is more tractable than in northern countries or in inclement seasons. Mr. Wallace makes the following remarks on diet.

"It will be highly necessary that the patient attend to the quantity and quality of his nutriment. All stimulating articles of diet, as also acids and ascescent and flatulent vegetable food, or whatever might disorder the bowels or disturb digestion, should be as much as possible avoided. The quantity of nutritious food should in general be less than the patient is in the habit of using. A small portion of wine or of his usual beverage may be allowed to preserve appearances; but such a quantity as would excite or hurry the circulation, is most studiously to be avoided. The process of digestion and a healthy state of the bowels, of individuals under the action of mercury, are often much promoted by the mastication and deglutition of grains of allspice or pepper occasionally during the day, and by covering the abdomen with two or three folds of flannel.

In case it should be of importance to the patient to remove that fetid breath which is in some measure inseparable from the anti-syphilitic action of mercury, this may be effectually done by those lozenges, containing a portion of chloruret of lime, which have been manufactured by my directions, for this purpose, at the establishment of Bewley and Evans of this city. These lozenges will also contribute to the prevention or removal of any tenderness of gums produced by mercury." 118.

We must say that we differ from Mr. Wallace. We have generally found a diminution of diet during a course of mercury prejudicial. We are not for stimulating patients; far from it. But we feel convinced, from experience, that in the majority of cases, a diminution of diet and the exhibition of mercury at the same time, are extremely likely to injure the health, and protract rather than expedite recovery, We always make it a point to ascertain what the patient has been accustomed to take in the way of food and of stimulus, and to put him as nearly as possible on his usual footing.

Mr. Wallace remarks that the case must be carefully watched, and alterations in the condition of the sore met by appropriate alterations in the local and general treatment. If the sore becomes inflamed or irritable, the mercury should be discontinued and antiphlogistics had recourse to, until those morbid states are removed. If, on the other hand, it becomes indolent, or increase in dimensions, we must endeavour to ascertain whether this arises from an insufficient or an excessive quantity of mercury, and increase it or withdraw it accordingly. Mr. Wallace very properly reprehends the hypothesis, that mercury cures the venereal disease by producing a kind of mercurial fever. The fact is, mercury answers best when it agrees with the local symptoms and the constitution. Mr. Wallace concludes this part of his subject by again reiterating his advice, that mercury be not exhibited whilst there is any inflammation or irritation. He generally precedes it by purgatives, or, if the patient is plethoric by the abstraction of twelve or eighteen ounces of blood. We have usually found salines and purgatives sufficient without venesection, and when we reflect that we are usually obliged to build up our patients after the second or third week of the course, we are led to pause before we unnecessarily deplete them in the first instance.

Mr. Wallace makes few observations on the disease in the female, as he says that its characters do not differ from those which it presents in similar structures in the male. In this we think that Mr. Wallace displays unprofitable brevity. We have always found the disease capricious, and sometimes more intractable in the female than in the male, but, at all events, it certainly presents very marked peculiarities, and is attended with many anomalies in the former. As Mr. W. has waived the subject we will not enter on it, but it is one on which there is much to be said.

Mr. Wallace makes some observations on the treatment of pregnant women affected with syphilis. There can be no question that under such circumstances, both the mother and the fœtus have the best chance of doing well, if mercury is cautiously and judiciously employed. Looking at the subject theoretically or practically, this would seem, and is really, the best practice, whatever has been urged to the contrary. But, if the disease be contracted shortly before parturition, the probable escape of the fœtus, and the approaching confinement of the woman, conspire to render delay advisable.

There are many curious facts to which Mr. Wallace has not adverted. We will mention a case in illustration of one. A pregnant woman had syphilis; but what the precise symptoms were, we cannot say. She was treated with mercury in the Lock Hospital of London, was apparently cured, and was discharged. Some time afterwards she was delivered ; the child, in two or three days after birth, became covered with a syphilitic eruption, and at the end of the third week it died. She has been pregnant twice or thrice since that time, and on each occasion the child has been attacked with the syphilitic psoriasis or lepra in a few days after birth, and died at the end of a fortnight or three weeks. The mother is a married woman, is apparently healthy, has neither discharge nor secondary symptom of any sort, and has not communicated any disease to her husband. Here we see a woman, to all appearance cured of the disease, producing successively infant after infant affected with it. As a contrast to this, we will mention another case. A woman, far advanced in pregnancy, was admitted into the same Institution, covered with that horrible eruption, half tubercle, half crust, which seems to be a compound of syphilis and cachexia, and which too often follows the improper use, or rather the abuse, of mercury. By sarsaparilla, and means of that description, she was greatly benefited, and left the hospital to be confined. She gave birth to a fine child, which has continued healthy. We might mention many other curious facts, and, indeed, we might readily consume our space in the detail of cases illustrative of the anomalies of syphilis in the female. It surprises us not a little, that Mr. W. has treated this part of his subject so lightly.

Mr. Wallace observes that females bear mercury worse than males, and that larger quantities of opium must usually be combined with it in the instance of the fair. We so rarely see syphilis in females in the upper classes, that we will not answer for them; but certainly the abandoned class of women in London bear mercury tolerably well.

Mr. W. observes that, if a woman contract the venereal during the state of lactation, that state is not, *per se*, an obstacle to the use of mercury. We would say that the child should certainly be put to nurse, or weaned, if a mother contracts the disease, as both this and the effects of mercury might be communicated through the medium of the milk. Menstruation is no impediment to the administration of mercury.

### Phagedænic Primary Syphilis.

Mr. Wallace uses the term phagedæna, as Mr. Abernethy has done, to express every form of destructive sore, whether owing to ulceration or sloughing. We do not think this a good arrangement, as phagedæna should be limited to that species of action in which there is a mixture or alternation of ulceration and of sloughing. If the latter preponderates greatly, it does not differ from mortification, and should be termed and considered such. To shew the impropriety of confounding these two states, we will merely cite two cases.

A young man had a sore in the groin, the result of neglected and mismanaged bubo. The edges of this were incautiously cut away, and stimulating dressings applied. The surface of the sore became of a yellowish ashen colour, the edges tumid, and presenting a superficies of slough, the part extremely painful. Day by day the sore became deeper, its surface always retaining the same appearance, the edges were also removed, increasing the diametrical extent of the ulcer, and at last the epigastric or the femoral artery (for it was not certain which of the two was implicated) gave way, and alarming hæmorrhage ensued. This patient was saved. The sore, when narrowly watched, was observed to increase, by a sort of succession of superficial sloughs, but still the ulcerative action was vigorous, and an eschar was never found in the wound. The sore was always on the increase, but this seemed as much the result of a process of absorption as of sloughing. This was phagedæna.

Another young man presented himself with the prepuce swollen, œdematous, and red, and a circular black spot was observed upon the glans. By the next morning half the glans was black, and on that evening the whole was so. In two or three days the glans fell into the pot, a putrid slough. A healthy sore was exposed, and in a week the patient was pursuing his usual avocations. This was mortification.

Surely these two conditions require separate denominations—surely a common term must create confusion. Mr. Wallace, however, employs a common term, and it is phagedæna. Let our readers recollect this.

We have yet to be convinced that there is such a thing as phagedænic primary syphilis. We shall see in what sense Mr. Wallace proposes it. He acknowledges, and this is an important admission, that phagedænic sores of the most opposite characters may present an exactly similar appearance at their origin; in fact, "that any of the forms of primary syphilitic phagedæna may commence by a pimple or a pustule; and that an excoriation, or abrasion, or wound, or ulcer of any form, or on any structure, or at any period of its progress, may assume a phagedænic character." This is all most true; and Mr. Wallace might have added, that any sore may assume a phagedænic character, not distinguishable from what is called syphilitic phagedæna, although there is not and cannot be anything approaching to syphilitic action in the case. In short, phagedæna is not a proper or specific, but a common action, and may occur, as inflammation may occur, in any kind of wound or ulcer. But we anticipate.

Under these circumstances, Mr. Wallace has not distinguished the varieties of phagedæna by the nature of their origin, but by the character of their progress.

"Phagedænic primary syphilis may extend either by ulceration, or by sloughing; and the sloughs may present a more or less white, or a more or less black colour. These are characters which can be easily ascertained, and according to them the SPECIES of these destructive sores may be classed. Again;—the state or degree of inflammation, and of sensibility or irritability attendant on phagedænic sores, is very different in different cases; and by these differences, which we can always appreciate, the VARIETIES of each species may be distinguished. It will also be sometimes necessary to take into consideration certain SUB-VARIETIES, resulting from age, habit of living, &c. &c." 131.

Mr. Wallace divides phagedænic primary syphilis into three species, and each species into three varieties—occasionally with sub-varieties. A sore may have a compound character; it must then be judged by the predominating and the worst. His species are these:—1. Without slough. 2. With white slough. 3. With black slough. Each species is divided into three varieties :—1. The simple. 2. The inflamed. 3. The irritable. So that we see the species is determined by the presence or absence of slough —the variety, by the presence or absence of inflammation or irritability.

### I. Phagedænic Primary Syphilis, without Slough.

The characteristic of this is the extensive or rapid destruction of tissue by ulceration. It comprises, in accordance with the preceding arrangement, three varieties :—The simple phagedænic ulcer—the inflamed phagedænic ulcer—and the irritable phagedænic ulcer. Mr. Wallace very properly observes that, strictly speaking, there are few phagedænic sores that have not some slough ; but in these the ulcerative action prevails.

#### A. *Simple Phagedænic Primary Syphilis without Slough.*

Its characters are extensive and sometimes rapid ulceration, without much inflammation or irritability. The surface is, in general, if not altered by mercury or other dressings, of a dusky yellowish white colour ; and very frequently serrated or nibbled at the margin and edge, which are often sharp and pependicular, or slightly undermined. The surrounding parts present a deep or livid red and swollen appearance, but there is not that circumscribed induration proportioned to its extent which is observed in the regular syphilitic sore.

"It sometimes exhibits a remarkable disposition to penetrate, rather than to extend along or over the surface. Hence it is, that when it occurs at the corona glandis, it often makes its way, if neglected or mismanaged, between the integuments and body of the penis, or else through the root of the prepuce externally ; and when it attacks the region of the frænum, it often penetrates so deeply as to open the urethra, although the ulcer may not in either case appear externally to be making much progress. On other occasions, this form of phagedænic primary syphilis will continue to extend its ravages at the circumference of the ulcer after the process of granulation has commenced, or is making considerable progress in the middle of the sore." 135.

When it attacks the prepuce or the glans, it sometimes rapidly destroys these parts without producing much pain. The constitution appears to sympathise greatly with this sort of ulceration. If it is at all rapid in its progress the patient becomes in a few days pale and languid, but he does not suffer so much local distress as might be anticipated. The following is the treatment recommended by Mr. Wallace.

"It is necessary, whenever we meet with a case of the simple phagedænic primary ulcer, if it has not been previously complicated by improper treatment, to subject our patient to a course of mercury,—regulated according to the principles formerly laid down ; and we shall always be gratified by the result. But if, on the other hand, the patient has been dabbling with mercurial remedies, and if there be reason to suppose that his constitution has been, in consequence, more or less disordered, we shall act more judiciously by suspending for a time the use of mercury ; and endeavour, by proper measures, but principally by attention to the mode of living of our patient, and by the use of the mineral acids with sarsaparilla, to restore the system to a state of tranquillity, before we enter again on mercurial treatment ; which may, however, be then used with success." 137.

The local treatment recommended by Mr. W. differs little from that pursued by him in the simple syphilitic sore. He has found fumigation of the part with the red sulphuret of mercury beneficial. Mr. W. observes, that in conducting the treatment of this form of sore, much caution, and attention to the rules already laid down, are necessary.

There is one circumstance to which we cannot forbear adverting. Mr. Wallace states that, this form of sore differs from the regular one of primary syphilis in little more than the severity and extent of the ulcerative stage. Now, it will be recollected that Mr. W. discountenanced the use of mercury in that stage of primary syphilis, yet here, where it is exaggerated, he preserves the employment of mercury. Logically this is an obvious inconsistency, but practical facts are superior to logical agreements.

In our observations on "the primary syphilis," we stated that we did not entertain much repugnance to the administration of mercury during its ulcerative stage, if certain precautions were attended to. There we went farther than Mr. Wallace. We must own that our experience, such as it is, leads us in the present instance not to go so far. If, as Mr. W. freely admits, any wound, abrasion, or sore may take on the phagedænic character, it is more than probable, it is almost evident that the character in question is accidental, that is, dependent on circumstances unconnected with the original virus. Now mercury is given for the original virus, and we cannot understand, as a matter of reasoning, why it should be "almost indispensable" in the treatment of that which is unconnected with the virus. Setting aside the reasoning, we are convinced of the contrary, as a matter of fact. We will mention a case or two in illustration.

A man was admitted into the Lock Hospital of London, with a circular syphilitic sore on the dorsum of the penis. He had been accustomed to live freely and to drink spirits and beer. When admitted he was purged, put on the broth diet of the hospital, and of course was deprived of his accustomed stimulus. In the course of a few days the sore began to spread with rapidity and had all the characters of phagedæna, without slough. He was immediately put on a generous diet, and allowed a pint of porter daily, and the sulphate of quinine was prescribed. In a few days the phagedænic action ceased, the sore became healthy, cicatrization commenced, and then, but not before, he was put on mercurial inunction. The sore healed rapidly.

Another patient was admitted into the same Institution, with a sore on the lower part of the glans and prepuce ; it had destroyed the frænum. It had commenced three months previously, and it had all the characters of a syphilitic sore, rather sloughy, and not likely to do well without mercury. Besides the sore, there was a small pustular eruption. This patient was a cook at an eating house, and had been accustomed to live well, and to take excessive quantities of ale and porter. He had merely taken some corrosive sublimate at the commencement of the complaint. Soon after his admission (May 13) he was ordered to rub in the mercurial ointment, and was allowed the broth diet of the house. On the 25th, the sore was extending rapidly by phagedæna on the glans and on the prepuce, and there was some disposition to bleeding. The mercury was immediately omitted, a pint of the decoction of sarsaparilla daily substituted for it, and the patient put on a very full meat diet, with a pint of porter daily. In two days the phagedænic action had ceased, and in five days (the 30th) the sore was more than half healed. This patient subsequently went through a mercurial course for the secondary eruption to which we have alluded.

We really do not exaggerate when we state, that we could crowd these pages with cases of this description, cases displaying phagedæna as evidently unconnected with the primary virus and dependent on external and fortuitous circumstances, cases proving as satisfactorily as cases can do so, that phagedæna *per se* does not require mercury, but on the contrary, is most generally aggravated by its employment. The two cases, however, which we have related, appear to us to be conclusive on this point, at all events we can dwell on it no longer. As we proceed with Mr. Wallace's account of the other species of phagedæna, we shall take occasion to enlarge upon the question.

B. *Inflamed Phagedænic Primary Syphilis, without Slough.*

This differs from the preceding in the degree and other characters of the surrounding inflammation and swelling. It is, according to Mr. W., seldom observed unless under circumstances of great neglect, and in persons of good constitution, though, perhaps, of dissolute habits. The degree of constitutional derangement is, in general, comparatively trifling, although the local inflammation may be great.

It may occur on any part, but the ulcers that more commonly assume this character are those situated on the internal surface of the prepuce, at the corona glandis, or at the side of the frænum; they almost constantly produce either phymosis or paraphymosis. When the former occurs, it is, of course, difficult to form a decided opinion on the nature of the sore. But Mr. W. observes that this information is not, on the whole, so important as it might seem to be, and therefore that the operation of dividing the prepuce is seldom necessary.

Inflammation may attack a primary ulcer during any of its stages, but most commonly it does so during that of ulceration; even if it occurs in that of granulation, ulceration is generally produced, so that inflamed phagedænic sore is rarely seen in a state of granulation.

Mr. Wallace observes, that the treatment of inflamed phagedæna consists in the abstraction of blood generally and locally, emollients, in short the antiphlogistic treatment, until the inflammatory action is subdued, and the sore brought to the condition of ordinary phagedæna, when the treatment adapted for that is to be substituted.

"It sometimes happens, when the inflamed phagedænic ulcer is seated at the corona glandis, that the matter is not discharged as fast as formed,—owing either to a tightened state of the orifice of the prepuce, which obstructs the escape of the matter, or to the tumefaction of the glans preventing the matter from coming forward. In either case an abscess is formed, which may open outwards at a point correspoding to the corona glandis, or the matter may burrow under the fascia of the penis, and extend even to the pubis, before it makes its way externally. In the former case, if the disease be mismanaged, the prepuce may be lost; while in the latter case, a most troublesome form of fistulous abscess may be produced, which will require many weeks to heal. These unpleasant consequences are, however, easily avoided by an early incision into the prepuce at the point corresponding to the abscess. The matter may in this way be freely discharged, and the opening will afterwards heal without much trouble. Should the matter have passed under the fascia of the penis, a larger opening will be required—otherwise the matter will still lodge; and although it may in such a case be partially discharged, the fistula will not heal, and a troublesome indurated state of the parts will result. When such an occurrence has unfortunately taken place, the disease must be treated according to those principles which will be hereafter laid down for the management of fistulous buboes, &c. &c." 143.

Mr. Wallace appears to have a great objection to slitting up the prepuce; when he does so, it is at the side of the frænum. We confess we do not participate in Mr. Wallace's repugnance to this operation, and, when we are fearful of mischief beneath, we make no hesitation of performing it. We have several times had reason to congratulate ourselves on having done this, and in more than one instance have we seen the let-alone practice followed by mischievous results.

In paraphymosis of recent origin, and attended with much inflammation, Mr. W. recommends that the patient be immediately bled, and that the process of reduction be assisted by making many minute punctures in the swollen prepuce. If this fail, the stricture must be divided; it is generally concealed between two tumid rings, one before, the other behind it, and mistakes are often occasioned by this circumstance. If the paraphymosis has been of many days' standing, it will sometimes be impossssible to reduce the prepuce, even after the stricture has been divided. This is owing to adhesions having been formed, and prolonged attempts at reduction are injurious, as well as ineffectual.

"It is not uncommon for a penis, which has a naturally short prepuce, or which presents a kind of congenital paraphymosis, to appear, merely in consequence of a great degree of subcutaneous effusion, to labour under a paraphymosis with stricture, when in reality no stricture exists. It is therefore important to inquire in every case of apparent paraphymosis, what may be the natural conformation of the parts affected. It should however be observed, that even when there exists a state of natural paraphymosis, the inflammation and tumefaction may cause a stricture by increasing the tightening, behind the glans, of the comparatively unyielding orifice of the prepuce; and it may then be almost as necessary to liberate the stricture, as when a common paraphymosis has taken place; but in such cases there is of course no use in making an attempt after the division of the stricture to bring the prepuce over the glans, as its natural length would not permit this to be done.

We sometimes find that the prepuce is not sufficiently long to cover the entire of the glans, and yet long enough to extend over the corona glandis; when this state of conformation exists, a kind of semi-paraphymosis or semi-phymosis may be produced, in which the seat of the stricture is neither behind nor before the glans, but about the middle of this part. When such an event occurs, it may be necessary to liberate the strictured part by an operation similar to that above recommended for phymosis." 147.

### C. *Irritable Phagedænic Primary Syphilis, without Slough.*

This is in general a consequence of mal-treatment, in peculiarly nervous or irritable constitutions. It is always extremely sensitive, particularly at night; when it is for the most part attended by such an increase of pain as to deprive the patient of rest. There is no proportion between the inflammation and the pain or sensitiveness attendant on it, for the latter far exceeds the former; yet there is not unfrequently a light, diffused, rose-coloured blush round the ulcer, which vanishes however on the slightest pressure. This ulcer appears sometimes very clean on its surface, and sharp at its edge and margin, as if cut by an instrument. On other occasions, its edge is more irregular or nibbled, as it were, and raised above the surface of the sore, but more rarely above the surrounding surface. The skin forming the margin of the areola, unless when it has been undermined, is never turned over, or rounded, as in some other forms of the primary syphilitic ulcer. There is little, if any induration of the base or circumference of the ulcer, and no disposition to regular granulation. In fact, for the most part the destructive process seems to go on steadily; the ulcer not exhibiting any steady disposition to the processes of reparation, or to those processes which seem to be ordinarily set up for the limitation of disease.

These ulcers frequently assume a herpetic character, healing on one side and spreading on the other. Mr. Wallace alludes to the difficulty of accounting for this curious circumstance; but he thinks that perhaps it may be owing to a law, "which seems to govern almost all organic actions, *viz.* that any given part loses, sooner or later, its disposition or power to continue any given morbid action, probably upon the same principle, that a given portion of soil will not support, for an indefinite period, any given vegetable production—not even a vegetable production to which the part may have been originally so suited, that it has been indigenous, or has sprung up in appearance spontaneously." We do not see the force of this suggestion. If Mr. Wallace alludes to morbid growths and morbid alterations of structure, we think the parallel not altogether just; for although it is true that the growth or the alteration do not, and apparently cannot, continue indefinitely, yet the tendency always seems to destruction, and not to reparation. If, on the other hand, Mr. Wallace alludes to the reparative processes, under ordinary circumstances, they do not account for these herpetic sores, as we see them regularly occurring on one side, and the actions of destruction as regularly progressing on the other.

Mr. Wallace observes that—

"When we observe an ulcer healing in one direction, and extending in another, we are disposed to presume that this must depend on a local cause; for as the influence of constitution would seem to be necessarily exercised equally on all parts of an ulcer, it does not appear, if all these parts be in the same state, how the same constitution could cause one portion to heal, and the other to ulcerate. We also sometimes observe in the same individual a healing ulcer on one part of his body, and an irritable spreading ulcer on another part. This is in fact a phenomenon, in some measure, of the same character as the former; and although we must presume, that in this case there exists also some local cause of the peculiarity, we are for the most part, if not always, ignorant of the nature of this cause. Such cases are however to be borne in mind, as they materially assist in explaining that combination of sores of different characters, which we sometimes observe to result on the same individual from the same contamination, and the occurrence of which has been used as an argument to prove that venereal diseases are not produced by a specific cause or morbid poison." 150.

We are disposed to devote some attention to this subject, for these herpetic sores are not very unfrequent, are always difficult of management, and not uncommonly end disastrously under injudicious treatment. Mr. Wallace appears inclined to attribute the peculiarity to a local cause. In this we differ from him totally. We believe that the cause is of a constitutional character; first, because it only occurs, so far as we have seen, in persons whose health is manifestly bad: secondly, because we have always found it yield to constitutional treatment: and, thirdly, because it is not uncommon to find two sores of this description, unconnected with each other, in the same individual.

We have had occasion to observe two species of this herpetic ulceration; in one the action is chiefly in the cutis; in the other it is manifestly seated in the cellular membrane, and the cutis is only secondarily affected. The latter has received, we believe, from some persons, the name of the "burrowing sore," but we do not think the term a good one, as, so far as we have seen, the sore has displayed a disposition to spread superficially rather than deeply, and the designation "burrowing" would convey an erroneous idea in this respect.

Mr. Wallace remarks that the constitutional disturbance is rather of the character of irritability than of fever. The pulse is in general quicker than natural, the sleep is often greatly disturbed, and there exists at times much thirst. The symptoms however are very capricious. In many respects we cordially agree with Mr. W. in the following observations upon mercury.

"Rest, emollients, anodynes, laxatives, together with a suitable regulation of diet, will often be sufficient to remove the morbid sensibility of the irritable phagedænic primary ulcer; and until this end be gained, mercury as a specific should be interdicted; for so far as I have had it in my power to make observations on the mercurial treatment of such sores, this medicine has always appeared to me to be injurious; and if persevered in, an increase of irritability, local and general, hectic and secondary symptoms of a very unmanageable kind may result. But as soon as the irritability of these ulcers is subdued, and the process of granulation has fairly set in, we may cautiously administer mercury, if, after having taken into consideration all the circumstances of the case, we deem it necessary." 151.

We think that the foregoing observations, brief as they are, are sufficient to shew that Mr. Wallace is a practical man. There can be no question that, as a general rule, mercury is pernicious in these herpetic sores; but there can also be no question that it is occasionally indispensable. As an illustration of this principle we will briefly mention the particulars of a few cases.

*Case* 1. A young man applied to us with ulceration of this character, extending uninterruptedly from near the anterior superior spinous process of the ilium to the anus. Added to this there was a similar sore, unconnected with the former, on the skin of the penis, near its præputial end. The spreading edge was in each the superior, the healing was the lower. The ulceration seemed rather in the cutis than in the subcutaneous cellular membrane. The patient was pale, feeble, much out of health, but not excessively emaciated. We need say no more of his history than this, that the ulceration had existed for five months, and he had been taking mercury for upwards of three. We put the patient on sarsaparilla, occasional purgatives, and the daily employment of the compound tincture of benzoin as a wash. With some occasional variations in the details, this treatment was pursued until the patient was quite well, which was about two months from our first seeing him. He was directed to live well.

*Case* 2. A man was admitted into the Lock Hospital with two rather sloughy-looking sores, but apparently of a syphilitic character, on the dorsum of the penis. These had existed for some time, and the patient had taken no mercury. He was sallow, and out of health; he had also been a spirit-drinker. Owing, apparently, to the diminution of diet on entering the hospital, the sores in a few days began to spread, coalesced, and assumed the herpetic character, travelling from the root of the penis towards its præputial end. The patient was immediately ordered sarsaparilla, generous diet, and porter. The sore was washed daily with the benzoin. His health improved, and the sore, though retaining its herpetic character, had a better appearance. From some cause or other the sarsaparilla was discontinued in a few days, and quina substituted for it. The sore continued to advance, and at the termination of three weeks it had proceeded from near the scrotum to the loose edge of the prepuce, and had completely encircled the penis; as it advanced it healed on the opposite border, so that it was really no larger at last than at first. The patient was now put on mercurial inunction and sarsaparilla at nearly the same time. The sore rapidly lost its peculiar character, and in ten days or a fortnight it was healed. The patient went through a mercurial course with sarsaparilla and a generous diet, and he has had no further bad consequences.

*Case* 3. A patient was admitted with darkish inflammatory swelling of the prepuce, and a sloughy sore on its extremity. There was also a bubo, which had ulcerated, in the left groin, and the skin was very dark, and likely to be destroyed. The patient's health was apparently broken down, he was pallid, thin, and so extremely nervous, that he burst into tears on the slightest occasion; the pulse was frequent, the skin rather warm. The history of the case was this:—A sore had appeared on the extremity of the prepuce about nine weeks previous to his admission. About a week afterwards the bubo appeared. He now rubbed in mercurial ointment for about ten days, when he caught the influenza and discontinued the inunction. The sore had healed but left induration. About six weeks previous to his admission the bubo broke, and phymosis became established. The præputial sore for which he applied had appeared about three weeks, and was attended with much pain and a rapid increase. He had been subjected to anxiety of mind, and compelled to exert himself in body. Since the inunction he had taken some oxymuriate.

The patient was put upon salines for a few days, when opium was given him at night, on account of sleeplessness, and quina with generous diet and wine prescribed. At first the præputial sore and the ulceration in the groin extended by sloughing, but after about a week's continuance of this tonic plan the sloughing process gradually ceased, and they assumed the herpetic character. The free prepuce was gradually eaten away till at last the patient was very neatly and very effectually circumcised. The spreading edge of either sore was the superior. After about two months the sore on the penis was quite healed, but that in the groin, though much diminished, continued, and still retained its herpetic character. This sore distinctly spread by successive sloughs in the subcutaneous cellular tissue, the cutis ulcerating subsequently. About the time that the sore on the penis healed, a tubercular eruption appeared on the body. The patient's health was greatly improved, he had gained flesh and strength, his appetite was good, and he slept without the assistance of opium. This was the time to put him upon mercury. He was directed to rub in about ten grains of the mercurial ointment every night, and when the gums became affected this quantity was diminished. The improvement in the sore was marked and rapid, and at present, this plan having been pursued for about three weeks, it is nearly healed, whilst the tubercular eruption is passing away.

We could give the particulars of some other cases, but our limits compel us to refrain, and these are enough for our purpose. Let us take a rapid glance at the facts which they disclose.

If we cast a glance on the preceding cases we shall observe the following facts, or rather we may form the following inductions. In all, the health was impaired, and that being the only common condition, may fairly be supposed to be the only common cause. In the first case the patient had evidently taken too large a quantity of mercury, and the cure was effected without its further employment. In the second case, mercury was indicated prior to the sore's assuming the herpetic character. This followed a diminution of diet and the abstraction of an accustomed stimulus—circumstances which are well known to be productive of injurious consequences after injuries, operations, &c. The adoption of the tonic plan did not arrest the spreading of the sore, but it improved the health, and then mercury (which had been indicated at the first) was beneficial. In the third case the particulars were nearly similar, with this exception, that mercury had not been withheld, but inefficiently administered in the first instance. The cases, considered in the whole, seem to prove that mercury is neither absolutely necessary nor absolutely prejudicial in cases of the herpetic phagedæna, but that its exhibition must depend upon the history and circumstances antecedent and concomitant. The herpetic character appears to be accidental; but if the case is a syphilitic one, the herpetic character itself seems indisposed to yield, except to mercury. Such, at least, is the conclusion which we have drawn from the preceding and from other cases.

We have not narrated any facts illustrative of the action of mercury, injudiciously administered, on these herpetic ulcerations. It is not because we have failed to witness such, for, unfortunately, we have seen this in several instances. The consequences have been most disastrous—extensive local destruction, and permanent constitutional injury. Nay, we have reason to believe that this form of sore was formerly very fatal, for no other reason, we suppose, than because mercury was lavishly and wildly given for it. Under any circumstances the sore is obstinate and difficult to manage, and perhaps there is none which more imperatively requires discrimination and judgment on the part of the surgeon.

We subjoin some further remarks on the treatment by Mr. Wallace. They appear to us to be characterized by accuracy and candour.

"The reader is not however to suppose that the irritable phagedænic primary ulcer is always so easily managed; for it must be admitted that it will sometimes resist the treatment above suggested. In these obstinate cases, great advantage will often be derived, after the preceding treatment has been employed, from causing a superficial slough along the edges, or on the surface of the sore, by the nitrate of silver; or from touching the edge and margin of the ulcer with the undiluted arsenical solution of Dr. Fowler, or with the strong nitrous acid.

But such remedies do not succeed so often in this form of irritable phagedænic sore, as in some others which will be hereafter considered. Indeed I have known them, when the ulcer was surrounded by a diffused areola, rather to promote than to stop the process of destruction; and to increase that peculiar appearance at the edge of the ulcer, as if a part had been cut out with a sharp instrument. In such cases we must try an opposite system, and we shall thereby frequently succeed in bringing about a healthy state of the ulcer. Thus we may employ a combination of the extract of opium with mercurial ointment in the proportion of a drachm of the former to an ounce of the latter. A dressing composed of the tr. opii with the mel. rosæ, and the liquor plumbi subacetat. will often be found a useful application. The balsams or turpentines will also occasionally succeed, when other means have failed. The uncertainty, however, with which all our remedies act in these and analogous cases, affords a strong proof among many others, of our very imperfect knowledge of those principles which should regulate the application of such agents.

In no case of the irritable phagedænic primary ulcer, are we to neglect the closest attention to the state of the system; for this is often of much more importance than local treatment. I have known some examples of this disease to resist obstinately every topical application, and to improve immediately from change of air. It may be said that the constitutional treatment of this form of disease consists chiefly in the employment of such remedies as will improve the general tone, and tranquillize the nervous system. For the former purpose, mineral acids, the sulphate of iron, the sulphate of quinine, and very minute doses of mercury to act on the secretions, are extremely useful; and for the latter, sarsaparilla, opium, Dover's powder, cicuta, or hyoscyamus. When there is reason to suppose that a state of nervous irritability is kept up by acidity in the *primæ viæ*, and this is not unfrequently the case, such medicines as the carbonate of ammonia, the aqua potassæ, or the aqua calcis, act with advantage. These remedies may be often usefully combined, and benefit derived from their combination, which could not be obtained from them separately. Thus the infusion of sarsaparilla in lime water is a most valuable medicine; calomel or blue pill combined with Dover's powder,—sarsaparilla with opium, cicuta, or hyoscyamus,—sarsaparilla with nitrous acid, &c., will be also found extremely useful; and in some cases, the most decided advantage may be obtained from the carbonate of ammonia with large doses of opium or henbane." 152.

Mr. Wallace finally remarks that the preceding varieties of phagedæna glide into each other, that a sore may be inflammatory at first and irritable afterwards, and vice versâ, and occasionally the same sore will display a combination of the characters of the others, &c.

### II. Phagedænic Primary Syphilis with White Slough.

This is a disease of frequent occurrence. Most syphilitic sores have a white sloughy matter on their surface, but those only belong to this head, which have caused or are causing extensive destruction.

#### A. *Simple Phagedænic Primary Syphilis with White Slough.*

This is perhaps more frequently the consequence of secondary than of primary syphilis. The surface of the sore is covered either by a stratum of white or yellowish-white sloughy matter, or by an incrustation formed by the desiccation or induration of this matter. It is a disease which occurs more frequently on the glans penis than on any other part, and in every situation it is accompanied by very little inflammation, and by still less irritability; but it is always surrounded by a greater degree of induration than any of the preceding varieties of phagedæna.

This disease may commence by the gradual conversion of a comparatively large portion of texture into a whitish slough, or it may begin in the same manner as any other syphilitic sore, and quickly assume its own peculiar characters; that is, any sore may assume the appearance of phagedæna with white slough. The texture of the slough is, in general, more or less stringy or tough. When exposed to the atmosphere it becomes more yellow, then brown, and lastly almost black. When dried it often forms a very hard crust on the sore.

Mr. Wallace remarks that this sore is always influenced in the most salutary manner by mercury; but that the latter if continued generally produces mischief. We must therefore be upon the watch, and if we find that mercury is losing its effects, or that the disease is becoming rather worse, we must instantly suspend it. The nitrate of silver is a good application, but if used too often, it is apt to occasion unpleasant consequences. Mr. Wallace has found large doses of sarsaparilla very useful when it is necessary to refrain from the use of mercury. It was its employment in these cases that first removed from his mind a doubt respecting the efficacy of this medicine. How a doubt ever got into Mr. Wallace's mind, is to us a matter of surprise.

From what we can collect, the sore to which Mr. Wallace has chosen to apply the foregoing denomination, is that which is often left on the separation of the crust of rupia, or of that of any of the varieties of the cachectic eruption. We would caution our readers against thinking so much of mercury, as even Mr. Wallace appears to do. This eruption and these sores are for the most part observed in those who have already been poisoned with mercury. How further quantities of that drug are to unpoison them passes our conception. There are many very curious considerations attendant on the treatment of this form of disease, and it certainly is a fact that small quantities of mercury are occasionally very beneficial. But we complain that Mr. Wallace appears to erect into the rule what we should deem the exception.

B. *Inflamed Phagedænic Primary Syphilis with White Slough.*

Any sore may assume this character, or the sore may present it from the first. In the latter case, it presents, when first noticed, a white slough surrounded by inflammation. This slough sometimes appears a little elevated above the surface, and may then be said to resemble somewhat a particle of curd of milk, having, perhaps, as much the appearance of being a deposition or secretion of lymph on an inflamed surface, as of a portion of the original structure converted into a white slough. These characters are often strongly marked at the corona glandis, or in the angle between this part and the frænum. The white matter or slough, by which this form of disease commences, quickly becomes excavated; and a sore is thus formed, which is lined by a white slough: the edge of the sore seeming also white, prominent, and surrounded by much and extensively diffused inflammation.

Mr. Wallace remarks that it is of great importance to distinguish the inflamed phagedænic sore extending by white slough, from that which extends by black. This should be done by examining the colour of the slough at its line of junction with the living parts, for in the centre the colour becomes altered by exposure, &c.

This inflamed variety of phagedæna is, like the other inflamed varieties of phagedæna, observed more frequently on the muco-cutaneous than on the cutaneous surface. The disease may occur on the cutaneous surface, and be covered with a crust; but this will not acquire much hardness, as the disease is attended with a great discharge, and spreads with much rapidity. When this variety of phagedæna is extensive, no matter where seated, its appearance is in general very formidable.

"It is remarkable that when this, or any other form of phagedæna, becomes very severe, if the patient has been previously threatened with any inflammation of the glands of the groin, their inflammation often decreases, or even ceases: and I have seen, under similar circumstances, the swelling and inflammation of the penis become less diffused, or more confined to the immediate neighbourhood of the disease. The induration, which may have surrounded the ulcer, also becomes much less. Hence it is that in this, as well as in some other of the phagedænic forms of primary syphilis, we do not observe that hardness, which has by many been considered characteristic of a venereal sore; but the degree of hardness attendant on venereal sores always depends very much on the nature of the adventitious actions, which happen to exist at the same time." 164.

The sore may advance with more or less rapidity, until it destroys a portion, or the entire of the glans or prepuce, when it often happens that a vessel gives way, and the hæmorrhage that ensues sometimes puts a stop to the disease.

When the sloughing process is about to cease, there is a marked diminution of the surrounding inflammation, and the quantity of sloughy matter at the same time decreases. Granulations now soon rise through the remains of this sloughy matter, while the surrounding redness becomes more circumscribed, and less intense, except at the very edge of the sore, where it may probably be increased, and thus forms a margin, in general about two lines in breadth. On the outside of this margin the skin after a time appears of a greyish or pearly-white colour, as if the part was callous; and from the inner edge of this red margin, a new skin proceeds, in general with great rapidity, in the form of a thin, smooth, and clear red covering,—soon followed by a contraction of the parts and perfect cicatrization.

The loss of substance produced by the sore is often less than might have been expected. When the muco-cutaneous surface of the penis is attacked, we are generally unable to observe its progress for any length of time, because phymosis is soon induced. On the external skin, its form of origin would appear, from the description of patients, to be just the same as that of common primary ulcer. On the corona, there is often a peculiar appearance of transverse fibres or laminæ, of a dark colour, exhibited at the centre or between the sides of the sore when they are drawn asunder.

The degree of constitutional disturbance is, for the most part, less considerable than in some other varieties of phagedæna, less formidable in their appearance. Fever, when it is present, has rather the inflammatory type.

The first indication in the treatment is the removal of the accompanying inflammation, which may be accomplished by the ordinary means. General bleeding, however, is seldom necessary. After the excess of inflammation has been subdued, we must treat the case upon the same principles, as those laid down for the management of primary syphilis.

"Having communicated my views respecting the treatment, under ordinary circumstances, of the inflamed phagedæna with white slough, I would beg the attention of the reader to an important fact ascertained by me, *viz.* that mercury, if given in full doses, or so as to produce rapidly its specific action on the system, will control, even in its most inflamed state, the progress of the phagedænic disease just described. This assertion may startle many of my readers, but it is no less true. It may scarcely experience a patient consideration from inveterate anti-mercurialists; but I trust that it will be put to the test of experience by the less prejudiced class of practitioners. I do not mean to say that the employment of mercury is a practice which should in such cases be ordinarily adopted, but I affirm that it will often afford the chief, if not the only instrument of protection to our patient, when this form of phagedæna is making havoc among parts of great importance. But, even then, its administration should be combined with such remedies as are suited to subdue inflammation,—perhaps with copious blood-letting, both local and general, with large nauseating doses of antimonials, &c. &c." 168.

Mr. Wallace has arrived at the conclusion that mercury, given in the cases of inflamed phagedæna that spread by a *white* slough, is beneficial, if administered to salivation. If, then, a case of phagedæna present itself, and if the phagedæna be making rapid progress by white slough in parts of importance, Mr. Wallace recommends the employment of mercury, whether there be much inflammation or not. If there be inordinate inflammation, antiphlogistics should be combined with the mercury. When the progress of the disease is arrested, the mercury should be intermitted, to allow the patient a breathing time; and, as soon as the system and the parts are tranquillized, the mercury may be resumed in a milder form. If mercury be employed uninterruptedly, "mercurial excitement or cachexia" is very apt to supervene.

"Whenever mercury is administered in cases of the inflamed white phagedæna, our attention should be unceasingly directed throughout the treatment to the character of the slough at its junction with the living parts; and should it change from white to black, in consequence of the intensity of the inflammation, or from any other cause; or should the inflammation increase while the patient is under the influence of mercury; or should the surface of the sore become clear of slough without any diminution of the inflammation and irritation; or lastly, should the system of the patient become deranged, while we find any extraordinary difficulty in exciting the mercurial action, the disease at the same time not improving, we must immediately intermit the mercurial treatment, as in such cases it will not serve any useful purpose, and if persevered in, may do infinite mischief.

It is to be here remarked, as a general rule, that a degree of inflammation, which would not prevent me from employing mercury, if that inflammation had existed previous to, or independent of, the action of this medicine, would be quite sufficient to deter me from using it, or would induce me immediately to intermit it, in case such inflammation had commenced while the patient was under mercurial treatment, or in a state of mercurial excitement or cachexia." 174.

Mr. Wallace makes some remarks on inflammation, which amount to little else than that inflammation sometimes is not inflammation, and that Mr. W. does not know what it is. These we may very well pass over. But Mr. Wallace compares iritis to phagedæna, and this we cannot pass over. How any man can see a similarity between these affections, is to us as incomprehensible as Johanna Southcotism. The one is a disease characterised by by an excess of the reparative action, the other by that of the destructive. Can any two things be more dissimilar. Mercury is useful in iritis; therefore, thinks Mr. W. it may be useful in phagedæna! We leave this to our readers.

We have given the more important portion of this section on phagedæna spreading by white slough. Are we converts to Mr. Wallace's doctrines? No. In spite of all his reasoning—in spite of his one fact—in spite of his false analogy between phagedæna and iritis, we are not converts; we doubt the soundness of his reasonings and the propriety of his treatment. We would not, however, stifle inquiry, and we, therefore, recommend the profession to endeavour to ascertain, whether the circumstance of the sore extending by a white slough is really indicative of the use of mercury. Limited as our space now is, we cannot detail the reasons which induce us to repeat, that we do more than doubt the propriety of Mr. Wallace's advice.

C. *Irritable Phagedænic Primary Syphilis, with white Slough.*

This is rare. When it exists, the sore often heals in one direction and spreads in another. The slough is, in general, more greenish than in either of the other varieties of white phagedæna, and the edges and margin are often reverted, or else undermined.

"The irritable white phagedæna, if it has not been previously mismanaged, is to be treated by short but full courses of mercury, combined with a large quantity of opium, or Dover's powder, or cicuta, or hyoscyamus. It will be useful in this form of disease to employ sarsaparilla in large doses. Much advantage will also be frequently obtained from the carbonate of ammonia, and the anodyne local treatment will succeed more frequently than any other form of dressing. I have, however, seen these sores, when the cutaneous margin was very livid and the edge sharp with great sensibility, much benefited by the application of escharotics to destroy the surface, or of powerful stimulants, as the arsenical solution, to excite to a new mode of action. It may be remarked, that the surface of the sores is uniformly easily destroyed by either the former or the latter class of applications. I have also obtained much advantage on some occasions from blistering the margin of their areola by rubbing it with the nitrate of silver." 180.

Mr. Wallace observes that the constitutions of patients, in whom this form of disease occurs, always require great attention, that relapses are frequent, recovery seldom uniformly progressive, and that the treatment which answers to-day may disagree to-morrow. Mr. W. concludes by observing, that it would appear that all the varieties of this form of phagedæna are, in general, favourably influenced by mercury. We know how difficult a subject this is, how cautiously general rules must be laid down, how contradictory even experience seems to be. Yet we cannot but dissent from Mr. Wallace's conclusion, and we really think that, though mercury, administered by a surgeon of judgment, well acquainted with the disease, and fully impressed with the dangerous character of the medicine he is employing, does in some instances prove highly beneficial, still we doubt the propriety of a general recommendation to its use, and we believe that, on the whole, there would result less mischief were it altogether banished from the treatment of phagedæna, than employed so freely as Mr. Wallace says it may be.

There is one great practical rule to which we always attend, and which, we believe, will save those who do attend to it from many blunders. Never be in a hurry to give mercury, but always improve the patient's health and strength before it is commenced. Of course the means must be adapted to the circumstances of the peculiar case; but, on the whole, the immense majority of patients with phagedæna require tonics, stimulants, and good diet. During the employment of these means, destruction seldom goes to any extent, and when the health is improved, we may begin to think of the propriety or necessity for the exhibition of the "specific."

It is rather a singular thing that we should differ thus from Mr. Wallace; but, whether the inconsistency is on our parts or his, we leave to others to determine. In the treatment of the ordinary form of syphilis, we are more mercurialists than he, adopt the mercurial treatment on more decided principles, and continue it for a longer time. But, in the instance of phagedæna, we do not go so far as he does—we do not consider mercury so useful, nor have we found it so safe.

In passing from the consideration of phagedæna without slough, or with white slough, or what is commonly called phagedæna, and entering on that of phagedæna with black slough, which is usually termed mortification, we cannot refrain from repeating our objections to the term phagedænic syphilis. We have said, and indeed Mr. Wallace acknowledges, that any form of venereal sore, the very slightest, may assume the phagedænic character. We will go farther than this, and assert that any kind of sore, independent of a venereal origin altogether, may take on that character in such a manner, as to render it indistinguishable from what is called syphilitic phagedæna. If this be true, and most surgeons of experience will acknowledge it to be so, we cannot understand why a common property should be considered a specific one. Our own impression and belief are, that phagedæna, *per se*, is in most cases to be treated on the same principles, and in the same manner, whether it occur in a syphilitic ulcer or a common one.

### III. PHAGEDÆNIC PRIMARY SYPHILIS, WITH BLACK SLOUGH.

Mr. Wallace divides this, like the preceding, into three varieties—the simple, the inflamed, the irritable. He observes that this division is highly necessary.

#### A. *Simple Phagedænic Primary Syphilis, with black Slough.*

The following are the essential characters of this variety. The slough is formed on the surface of the sore rather than at the edge, and the latter may remain quite free from the sloughing form of destructive action, while this process is extending on the former. This disease is accompanied by much greater induration of the surrounding parts than any of the other forms of phagedænic primary syphilis; but exhibits scarcely any inflammation, and is attended by very little sensibility. The sloughing process is also more slow than in the other varieties of the black phagedæna, and the slough frequently presents a diversity of tint, being sometimes of a much lighter colour than at others. It is in fact often of a tawny nut-brown colour rather than black, while the surrounding parts are often of a callous, or whitish-red colour,—depending apparently on a state of great induration, produced by an interstitial deposition round the diseased surface.

This form of phagedæna may attack the regular "primary syphilis," or the superficial varieties. It may occur in any situation, but perhaps most commonly on the corona glandis, where it often causes a very deep brownish-black sloughy ulcer, with great surrounding induration. It may commence during any stage of an ulcer; but it is most frequent during that of destruction. It is not uncommon among the out-patients of a hospital.

"This variety of phagedænic primary syphilis, notwithstanding its peculiar and alarming appearance, requires scarcely any form of treatment, either local or constitutional, different from that of the regular primary disease. In fact, the same mode and the same order of treatment may with the best consequences be employed in the one, as have been laid down for the other. I admit that a well-regulated course of evacuants and emollients, with quietness, &c., if persevered in, will in these cases as in almost all the other forms of primary syphilis, retard the progress of the disease, and perhaps induce healing actions. I have also observed, that these cases are very often extremely benefited by the internal employment of either the nitrous, or the nitro-muriatic acids; but the actions of reparation and perfect cicatrization may be produced in them with much more certainty, in a much shorter time, and with less expense to the constitution, by a mild and well-regulated course of mercury, than by any other means." 185.

This is so totally foreign to our experience, that we merely record Mr. Wallace's opinions, and pass on. We will merely add, that we would not, for a good round sum, treat this sore attended with black slough, in other words mortification, by mercury. We do not quarrel with Mr. Wallace's practice, but we would not follow it on any consideration.

Mr. Wallace, indeed, admits that the constitutions affected with this description of sore, often bear mercury ill. Escharotics also easily destroy the surface, and they are easily excited by stimulants. Mr. W. also observes that, where large and long-continued doses of mercury cannot be borne, it will often be of great advantage to interrupt the medicine occasionally.

B. *Inflamed Phagedænic Primary Syphilis, with Black Slough.*

Mr. W. observes, that this occurs under very different circumstances, but under all is uniformly aggravated by the employment of mercury. To contrast this with the former, which he avers to require mercury, he has opposed their characters in two columns, as under.

| "SIMPLE BLACK PHAGEDÆNA. | INFLAMED BLACK PHAGEDÆNA. |
|---|---|
| 1. *Attended by little or no inflammation.* | 1. *Attended by great inflammation,* |
| 2. *Attended by great induration.* | 2. *Attended by little induration.* |
| 3. *Attended by little tumefaction or œdema.* | 3. *Attended by great tumefaction or œdema.* |
| 4. *Sloughs principally by the surface.* | 4. *Sloughs as much at the margin and edge as on the surface.* |
| 5. *Slough of a brownish black colour.* | 5. *Slough of a deep black colour.* |
| 6. *Slow in its progress.* | 6. *Rapid in its progress.* |
| 7. *Not attended by great pain.* | 7. *Often attended by great pain.*" 187. |

Mr. W. remarks that the sub-varieties differ greatly from each other in many respects.

1. When gangrene occurs in robust persons, Mr. W. supposes that there be some predisposing cause besides the violence of the inflammation. In general, the patient has been guilty of neglect, or has led an irregular life, or has been injudiciously treated by mercury. This form of phagedæna may supervene on any sore, or the sore may display it from the first. It is not observed now so often as formerly, probably because mercury is not so indiscriminately employed. It presents various degrees of intensity, sometimes causing the rapid destruction of the entire penis, preceded by more or less violent inflammation; at others, being confined to the edge, or a portion of the surface of the sore. It then produces along the edge a sort of punctuated appearance,—the points being minute portions of gangrened structure. These black points, by increasing in number, often form one continued gangrenous line, surrounded by an intensely red and diffused areola. When the sore is in this state, very little indiscretion will almost certainly cause a rapid increase of gangrene.

Like all other forms of phagedæna, this frequently commences at the corona, and is then soon followed by much swelling and inflammation, with either phymosis or paraphymosis, according to circumstances. In the former case, we soon lose the opportunity of observing the surface of the sore, and if the disease increases, the prepuce continues swelled and often red, with a copious discharge of grumous bloody pus from its orifice. After a time, the portion of integuments corresponding externally to the sore, becomes still more swelled and more red; and if the progress of the disease be not controlled, the central portion of this red and tumid part loses its vitality, and changes into a black slough, which extends more or less; and at last, giving way to the pressure from the inside, the glans protrudes through the opening. It now sometimes happens that the glans, being constricted after its protrusion by the border of this opening, becomes strangulated and sloughs; but, more frequently, the sloughing process of the prepuce continuing to extend, the opening through which the glans has been protruded is thereby enlarged and all pressure removed. The glans is thus entirely uncovered, and the process of destruction often advances, so as to cause not only a kind of circumcision, by removing the entire of the prepuce, but also the destruction of more or less of the penis and scrotum. Often, however, the frænum and immediately contiguous part of the prepuce remain.

The pain attendant on the disease in robust habits is often very severe, and the constitutional disturbance is proportioned to its increase. In the commencement there may be little general derangement, but when the disease has advanced there is much symptomatic fever, and this is succeeded by symptoms of a more nervous character, which may end in the patient's death.

The treatment recommended by our author consists in the combination of evacuants with anodynes and with local emollients. He thinks it will be almost uniformly prudent to take away blood from the arm, and perhaps locally by leeches. Purgatives and emollients, and, after the evacuations, anodynes with antimonials in full doses, enter also into our author's list of remedies. Mr. W. remarks, as all have done, that a spontaneous hæmorrhage is often succeeded by rapid improvement.

Frequently on the cessation of the sloughing action, the eschar separates very quickly, and the process of cicatrization is remarkably rapid. On other occasions, when the reparative process has advanced a certain way, the sore often assumes an unhealthy appearance, ulceration again commences, and the disease may again advance with rapidity.

When the ulcer heals gradually and uninterruptedly, Mr. Wallace recommends that no mercury be given, the sloughing process having probably destroyed the specific poison.

When the reparative process is interrupted, we should endeavour to ascertain the cause of this, whether constitutional disorder, or error in local applications. Having ascertained the cause we should endeavour to rectify it. If, however, we can detect no cause, and if we cannot re-induce healthy action, it is probable that secondary symptoms are about to make their appearance. Are we to suppose that they will do so, and give mercury in consequence, or are we to pause? In the following sentiments we cordially agree with Mr. Wallace.

"Upon this point practitioners do not seem to be generally agreed; but upon my mind there does not remain any doubt that it will be more prudent to refrain from the employment of mercury; for it will generally happen that mercurial treatment will not in these cases control the occurrence of secondary symptoms. On the contrary, if this medicine be exhibited, secondary symptoms will sometimes appear even while the patient is under its influence; for the state of constitution then existing would seem to prevent this medicine from producing that effect, which is necessary to control the influence of the venereal poison; and if mercury be administered in such a constitution, it will, almost inevitably, not only fail to exercise a controlling influence over the effects of this poison, but also render them more obstinate and more severe in their character. This has always seemed to me, as already observed, to be the true explanation, why the symptoms of the venereal disease sometimes return or become aggravated, while the patient is under the action of mercury." 193.

Mr. W. employs, in such cases, sarsaparilla and nitrous acid, and waits for the secondary symptoms to appear before he treats them. These cases must be treated with judgment, and according to circumstances. We are under the necessity of passing over some very judicious observations on phagedæna in those accustomed to spirit-drinking, and can merely allude to the herpetic black phagedæna.

"In the herpetic form of irritable black phagedæna, as well as in that form which is not attended by a diffuse areola, I have very frequently derived the most remarkable advantage from pencilling the edge of the sore with Fowler's solution of arsenic, undiluted. I think I have also seen advantage from its use in cases which were attended by a diffuse areola; but of this I am not so certain. Its application causes in general very considerable pain at the moment, and for some time after; but I have scarcely ever used it in any case, in which the patient did not wish for its repetition, so great is the relief which follows in a few hours after it has been applied; and I have often succeeded by its means in producing a healing action of these sores, after innumerable other remedies had been tried in vain. In cases of a similar kind, I have also sometimes obtained signal advantage from cauterizing the diseased surface with strong nitrous acid; and on two or three occasions there was marked benefit produced by the actual cautery applied along the edge and margin of the sore. It should also be mentioned, that I have sometimes seen the nitrate of silver increase this disease, when applied as an escharotic; and this has occurred in cases in which the application of the nitrous acid has had a beneficial effect.

The constitutional remedies most to be depended on in all the forms of the irritable black phagedæna, are change of air and of occupation, opium, Dover's powder, cicuta, hyoscyamus, sarsaparilla in very large quantities, guaiacum, the carbonate of ammonia, and sometimes the sulphate of quinine." 207.

Mr. W. observes, that it would be vain to attempt the cure of these sores without constitutional treatment. He dwells, however, on the circumstances of extension by a white or a black slough, prohibiting it in the latter case, encouraging it in the former, and observing that in compound cases we must be guided by the relative quantities of the white slough and the black. Mr. Wallace here, as elsewhere, lays such stress on this, that it is evident he depends very greatly upon it, as a diagnostic mark of great importance. And yet we are very far from being convinced by Mr. Wallace, and, amongst other reasons, for this. We had heard that Mr. Wallace had laid down the distinction, and that he relied very greatly upon it. We were induced, in consequence, to watch some cases that presented themselves to our observation, and they seemed to oppose his views. To one of those cases we have already alluded. It was one of phagedænic ulceration in the groin; it spread distinctly by a white slough, yet that case was cured by a bottle of wine per diem. In another case, which we particularly remember, a young lad was admitted with black slough of the glans of the prepuce. The black slough separated, but some further extension by white slough occurred. This patient was rapidly cured by a bleeding to six ounces in the first instance, and the administration almost immediately afterwards of carbonate of ammonia and port wine. We could mention some other cases, but we would entreat our readers to attend to Mr. Wallace's suggestion, and judge for themselves. We repeat, that our own impression is against Mr. Wallace, but we have not sufficiently investigated the subject to have yet made up our minds.

From what we have already remarked on particular passages, our readers may presume that we are essentially opposed to Mr. Wallace on the subject of Phagedæna. We are so, and why we are so we need not reiterate now. We doubt whether the very nice distinctions of phagedæna into nine varieties will ever serve in practice. There is, in some measure, a difference of treatment for each, a very vital difference in this respect for several, and yet Mr. W. admits that they run into one another, are vicariously interchanged, and their characters are frequently composite and combined. Under such circumstances we feel convinced that attention to the minute rules of Mr. W. would lead to wavering, uncertain, and confused treatment; we feel assured that, instead of diminishing the difficulties now experienced in the treatment of phagedæna, it would augment them. Mr. Wallace, after all, makes mercury the rule for phagedæna. We would make it the exception.

We fear we must stop here. Our analysis has been already too long to allow us to venture on the remaining portions of the volume. These we must defer until our next number, when we hope to conclude the review of the work.

We have freely indulged in criticism, because we think that on such subjects, a reviewer, if qualified to give an opinion, has a right to do so. At the same time, we would beg to disclaim all dogmatism, and to confess that reviewer and reviewed must both be judged by the common sense and the experience of the public. If we have differed from Mr. Wallace we have given our reasons for doing so, and the length of these notices implies our conviction of the merits of their subject. We venture to say that, right or wrong, there are few works of the present day that bear the impress of more patient observation, and more careful consideration than does this of Mr. Wallace. It is highly creditable in every respect.

